# Review of the BCI Competition IV

**DOI:** 10.3389/fnins.2012.00055

**Published:** 2012-07-13

**Authors:** Michael Tangermann, Klaus-Robert Müller, Ad Aertsen, Niels Birbaumer, Christoph Braun, Clemens Brunner, Robert Leeb, Carsten Mehring, Kai J. Miller, Gernot R. Müller-Putz, Guido Nolte, Gert Pfurtscheller, Hubert Preissl, Gerwin Schalk, Alois Schlögl, Carmen Vidaurre, Stephan Waldert, Benjamin Blankertz

**Affiliations:** ^1^Machine Learning Laboratory, Berlin Institute of TechnologyBerlin, Germany; ^2^Department of Brain and Cognitive Engineering, Korea UniversitySeoul, Korea; ^3^Faculty of Biology, Bernstein Center Freiburg and University of FreiburgFreiburg, Germany; ^4^Institute of Medical Psychology and Behavioral Neurobiology, University of TübingenTübingen, Germany; ^5^Ospedale San Camillo, Istituto di Ricovero e Cura a Carattere ScientificoVenezia, Italy; ^6^MEG-Center, University of TübingenTübingen, Germany; ^7^Center of Mind/Brain Sciences, University of TrentoTrento, Italy; ^8^Institute for Knowledge Discovery, Graz University of TechnologyGraz, Austria; ^9^Swartz Center for Computational Neuroscience, Institute for Neural Computation, University of California San DiegoLa Jolla, CA, USA; ^10^École Polytechnique Fédérale de LausanneLausanne, Switzerland; ^11^Department of Bioengineering, Imperial College LondonLondon, UK; ^12^Department of Electrical and Electronic Engineering, Imperial College LondonLondon, UK; ^13^Physics, Neurobiology and Behavior, Medicine, University of WashingtonSeattle, WA, USA; ^14^Institute for Neurophysiology and Pathophysiology, University Medical Center Hamburg-EppendorfHamburg, Germany; ^15^Department of Obstetrics and Gynecology, University of Arkansas for Medical SciencesLittle Rock, AR, USA; ^16^Brain-Computer Interface R&D Program, Wadsworth Center, New York State Department of HealthAlbany, NY, USA; ^17^Department of Neurology, Albany Medical CollegeAlbany, NY, USA; ^18^Department of Neurological Surgery, School of Medicine, Washington UniversitySt. Louis, MO, USA; ^19^Department of Biomedical Engineering, Rensselaer Polytechnic InstituteTroy, NY, USA; ^20^Department of Biomedical Sciences, School of Public Health, State University of New YorkAlbany, NY, USA; ^21^Institute for Science and Technology AustriaMaria Gugging, Austria; ^22^Sobell Department of Movement Neuroscience and Movement Disorders, Institute of Neurology, University College LondonLondon, UK; ^23^Neurotechnology Group, Berlin Institute of TechnologyBerlin, Germany

**Keywords:** brain-computer interface, BCI, competition

## Abstract

The BCI competition IV stands in the tradition of prior BCI competitions that aim to provide high quality neuroscientific data for open access to the scientific community. As experienced already in prior competitions not only scientists from the narrow field of BCI compete, but scholars with a broad variety of backgrounds and nationalities. They include high specialists as well as students. The goals of all BCI competitions have always been to challenge with respect to novel paradigms and complex data. We report on the following challenges: (1) asynchronous data, (2) synthetic, (3) multi-class continuous data, (4) session-to-session transfer, (5) directionally modulated MEG, (6) finger movements recorded by ECoG. As after past competitions, our hope is that winning entries may enhance the analysis methods of future BCIs.

## 1 Introduction

Brain-computer interfacing (BCI) is an approach to establish a novel communication channel from men to machines. The crucial idea is to directly tap the communication at its very origin: the human brain. BCI technology is used to date primarily for intentional control. This branch of BCI research aims at the (partial) restoration and rehabilitation of lost functions in paralyzed patients (Kübler et al., 2001; Wolpaw et al., 2002). The focus of the fourth BCI competition was on BCI systems that are based on the motor and sensorimotor system of the brain. In line with the past three BCI competitions, this fourth BCI competition strives to help the field of BCI prosper by eliciting solutions for hard data analysis problems appearing in current BCI research.

Apart from communication and control, recently more and more alternative applications of BCI technology are being explored (Blankertz et al., 2010). These include enhancement of human performance (Haufe et al., 2011) and assessing subconscious perception (Porbadnigk et al., 2010, 2011). Data from those recent developments have not yet been included in the BCI competitions, but may pose interesting and novel challenges for future competitions.

### 1.1 Relevance of BCI competitions

**Figure 1:**
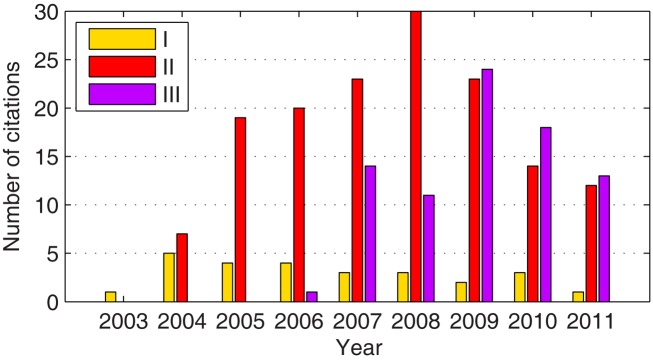
**Citations of the overview articles on previous competitions**. The histogram shows how many times the editorial articles on BCI competitions I (Sajda et al., 2003), II (Blankertz et al., 2004), and III (Blankertz et al., 2006) have been cited in ISI-indexed journals. Data were retrieved from the ISI Web of Knowledge on December 1st 2011.

**Figure 2:**
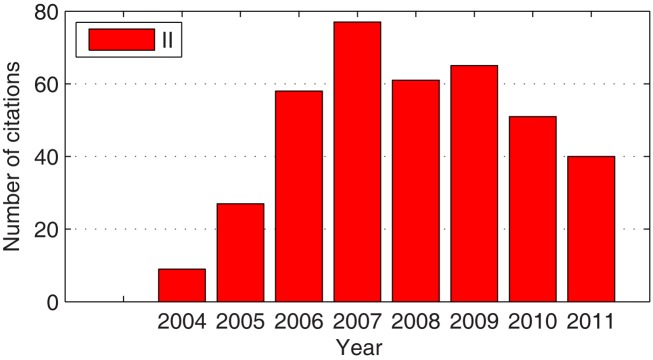
**Citations of the articles by the competition winners**. The histogram shows how many times the articles of the winning teams of BCI competition II (describing the winning algorithms) have been cited in ISI-indexed journals. Data were retrieved from the ISI Web of Knowledge on December 1st 2011.

The impact of the past three competitions on the field of BCI research is manifold and thus worth a closer look. One indicator of the overall relevance of the BCI competitions for the scientific community is the number of citations. Figure 1 shows how often the three overview articles on the past BCI competitions I (Sajda et al., 2003), II (Blankertz et al., 2004), and III (Blankertz et al., 2006) have been cited in ISI-indexed journals and conference proceedings. The overall sum is 255. From competition II on, the concept was introduced to have publications of all winning algorithms within one issue of a journal. This worked very well in the BCI competition II where all winner articles have been published in volume 51 of *IEEE Trans Biomed Eng* (Blanchard and Blankertz, 2004; Bostanov, 2004; Kaper et al., 2004; Lemm et al., 2004; Mensh et al., 2004; Wang et al., 2004; Xu et al., 2004). Such concerted publication leads to good visibility, and as a consequence to substantial citations, see Figure 2. (In competition III only some winning algorithms were published spread cross several journals; Wei et al., 2006; Galan et al., 2007; Zhang et al., 2007; Rakotomamonjy and Guigue, 2008.)

Moreover, research groups that are relatively new to the field of BCI can attract attention and get renowned if the performance of their algorithms is independently validated through the competition process. This is an attractive opportunity even for researchers who do not have access to an acquisition device for brain signals or a fully running BCI system. Additionally, some researchers of the better performing teams were hired or hosted by BCI groups (in particular the one contributing data sets to the competition).

Most important, the results of the BCI competitions provide an indication of what type of methods are effective. A good example of such a lesson that can be learned from the competitions is that common spatial pattern analysis (CSP/CSSD; Koles, 1991; Ramoser et al., 2000; Blankertz et al., 2008b) and its variants are a robust tool for exploiting ERD/ERS effects (Pfurtscheller and da Silva, 1999): Almost all data sets throughout all BCI competitions in which CSP was reasonably applicable (e.g., for multi-channel recordings or for paradigms in which differential ERD/ERS effects are expected) have been won by an algorithm involving a variant of CSP: competition II (2a, 4); competition III (1, 3, 4a, 4c); competition IV (1, 2a, 2b). The success of the CSP-based methods in the BCI competitions may have a promoting factor for the flourishing development of variants of CSP analysis (Lotte and Guan, 2011; Nikulin et al., 2011; Sannelli et al., 2011).

In contrast, the application of principle component analysis (PCA) or independent component analysis (ICA), which are very successful preprocessing methods in other application fields, seem to be a less effective ingredient to improve the classification performance in BCI (but note, that ICA was used in Xu et al., 2004). This advance of CSP compared to PCA and ICA may to a large extend be explained by the different strategies concerning the use of class labels. While CSP exploits the information contained in the labels in a supervised manner, ICA and PCA are unsupervised methods.

In this context, we would like to stress that the competitions are by no means a systematic evaluation of all available algorithms. Therefore, we would still like to encourage to explore the full realm of signal processing and pattern recognition algorithms for BCI.

### 1.2 The role of open data

BCI research is complex, and to design an online BCI experiment or successfully run a BCI application involves the cooperation of specialists from various disciplines. The availability of BCI data from past competitions is an important contribution to stimulate the interdisciplinary engagement of students and researchers from neighboring research areas, who can enrich the field of BCI. This is especially true for scientists specialized in signal processing, data analysis, and machine learning, but also for researchers from the field of human-computer interaction (HCI). While these specialists have the potential to improve the progress of BCI with new algorithmic methods or improved usability of BCI applications, the field of BCI needs to provide the fuel, that is data. Data, that on the one hand is typically noisy, high-dimensional, shows non-stationary characteristics, and thus provides a challenging test ground especially for the signal processing and machine learning community. On the other hand, BCI data represents – if interpreted as a signal for communication and control – an inherently unreliable and slow communication channel. From the viewpoint of HCI, the field of BCI can be considered a challenge as it requires highly robust interaction models in order to cope with the above mentioned challenges. Finally, any success story for the interaction design in BCI might be transferable into the other fields like usability of mobile devices or gesture controlled applications, which share some of these interesting characteristics.

### 1.3 Notes on the use of BCI-competition data

Despite of a number of high quality algorithmic solutions proposed by the competition winners in the following sections, the actual learning problems posed in this competition are surely of interest in the future, and the proposal of new methods for their solution can enhance the field of BCI. For this reason, the competition data sets have been provided online as open data. Furthermore, the labels of the test data, which have not been available to the participants of the BCI competition IV, have been published in addition.

We would like to encourage the use of this data and the publications of any results and insights. Upon publication of such results, however, we would like to draw your attention to three important aspects:

First, any performance improvement over the competition results, should be reported with a note of caution, as it could merely reflect random fluctuations. Ideally, the performance should be reported for larger amounts of test data.

Second, a comparison with the performance of the competitors should be drawn carefully only, as any post-competition work on the same data has been performed under the advantage of knowing the competition outcome, knowing the specific shortcomings of the submitted algorithms, and having insight into which classes of algorithms perform better or worse on that data.

Third, even so the test data labels are publicly available now, their use should be restricted to finally determine the performance of a method. The test data should not be touched at all during the algorithm design process and the determination of hyperparameters, as this can lead to a substantial amount of overfitting (Lemm et al., 2011).

### 1.4 Relevance of the data sets

For an overview, a list of data sets and the corresponding winning teams is summarized in Tables 1 and 2.

**Table 1:** **Overview of the data sets of BCI competition IV**.

#	Lab	# Channels	Paradigm and challenge
1	Berlin	64 EEG	2-Class motor imagery, uncued classifier application
2a	Graz	22 EEG	4-Class motor imagery, continuous classifier application
2b	Graz	3 EEG	Motor imagery, session-to-session transfer and eye artifacts
3	Freiburg	10 MEG	Decoding directions of finger/hand/wrist movements
4	Seattle/Albany	64 ECoG	Discrimination of movements of individual finders

**Table 2:** **This Table lists the winning teams for all competition data sets**.

Data set	Research lab	Contributor(s)
1	Institute for Infocomm Research, Singapore	Zhang Haihong, Ang Kai Keng, Guan Cuntai, Wang Chuanchu, Chin Zheng Yang
2a	Institute for Infocomm Research, Singapore	Kai Keng Ang, Zheng Yang Chin, Chuanchu Wang, Cuntai Guan, Haihong Zhang, Kok Soon Phua, Brahim Hamadicharef, Keng Peng Tee
2b	Institute for Infocomm Research, Singapore	Zheng Yang Chin, Kai Keng Ang, Chuanchu Wang, Cuntai Guan, Haihong Zhang, Kok Soon Phua, Brahim Hamadicharef, Keng Peng Tee
3	Biomedical Signal and Image Processing Laboratory (BiSIPL), Sharif University of Technology, Tehran, Iran	Sepideh Hajipour, Mohammad Bagher Shamsollahi
4	Cortex Team, Research Centre INRIA, France	Nanying Liang, Laurent Bougrain

The BCI competition fosters algorithmic solutions, which allow for a single-trial assessment of mental states. For the neurosciences, such developments in signal processing and machine learning are clearly relevant as these single-trial data analysis methods provide a possibility to monitor the acting and behaving brain. This is a prerequisite to study the dynamics of brain processes, and eventually develop new reactive experimental paradigms, that vary, e.g., stimulus conditions depending on the current state in a closed loop.

The data sets of this competition all deal with motor paradigms, and more specifically with oscillatory signals which are related to imagined motor actions or motor execution. As an example, direct clinical relevance of BCI technology can be expected for the support of rehabilitation training in patients suffering from stroke (Silvoni et al., 2011) in cortical motor areas. However, as changes of oscillatory processes are not uniquely observed during motor activities, but represent a rather general high-level characteristic of many brain processes, the benefit of this BCI competition should extend from motor system research to other fields.

Data set 1 of the BCI competition IV addresses the challenge to correctly deal with intended non-control periods and uncued periods of control activity. This is of high clinical relevance, as any practical application of a motor imagery BCI system will require that the BCI system recognizes periods of resting and coming back to active BCI control.

Data set 2a enlarges the number of control classes from two to four. Compared to the simpler setting of only two motor imagery classes, this enlargement contains the risk of a reduction in classification accuracy. However, it also offers the potential of higher information transfer rates, and more natural interaction paradigms between user and application. In combination with the continuous classification setting, this is clearly of practical relevance.

Data set 2b challenges the session-to-session transfer of classification models. Avoiding the time-consuming re-calibration of the BCI system, such approaches are of high practical importance for end-users, who want to use a BCI on a daily basis.

Data set 3 is a collection of magnetoencephalography (MEG) signals. While most motor paradigms in non-invasive BCI make use of the lateralization of motor-related signals (e.g., ERD/ERS effects over the left hand and right hand cortex), this data set seeks to extract a multi-class decision from a single hand only. Comparable to data set 2a, the expansion from two to more classes has the potential to boost the information transfer rate of a BCI. Furthermore the data set is an example for the possibility to infer hand movement directions not only from single cell spiking activity (e.g., by intra-cortical single unit recordings; Georgopoulos et al., 1982; Velliste et al., 2008), which are known to realize a directional coding, but also from non-invasive measurements of larger populations (Waldert et al., 2008). Despite of its practical restrictions (an MEG system is neither practical nor affordable for patients), this MEG-BCI could still be applied in conjunction with online feedback, e.g., for stroke rehabilitation attempts (Buch et al., 2008) or for prosthesis training.

The goal for data set 4 of the BCI competition IV was to infer the flexion of individual fingers from signals recorded from the surface of the brain via electrocorticography (ECoG). Determining the relationship of ECoG signals with finger flexion provides new neuroscientific understanding, and may eventually lead to improved brain-computer interface systems.

### 1.5 Overview of the article

After some general remarks concerning the concept and the BCI competitions in Section 2, the subsequent five sections, will characterize each data set contained in the BCI competition IV in detail, including an assessment of its relevance to the field, experimental details, the data format, the applied evaluation criterion for submissions, and a brief outcome. The article closes with a section about the overall results of the competition and a discussion. The latter includes prospective topics of subsequent competitions.

The winning labs published individual articles on their approaches, see (Ang et al., 2012; Flamary and Rakotomamonjy, 2012; Sardouie and Shamsollahi, 2012; Zhang et al., 2012).

## 2 General Structure of the Data Sets and the Machine Learning Task

Challenges posed within the BCI competition typically contain a problem description, a training data set, a test data set, and a description of the evaluation metric that is applied to determine the performance of contributed algorithms.

### 2.1 Training data

This collection of data (also called calibration data) comprises the data epochs from EEG, MEG, or ECoG recordings, the labels or markers that describe the tasks that were to be performed by subjects at recording time, and the cues which had been presented to them. In addition, the groups providing the training data describe the specific performance metric according to which any participant’s competition entry will be rated. Participants used this information to develop a processing method that was able to estimate labels based on data. Any method could only be successful, if it generalized well on new test data.

### 2.2 Test data

This data set (also called evaluation data) contains data epochs, but no labels or markers. The labels do exist but were secret to the participants. The task of participants was to estimate the labels of the test data and send them in. The data providing group evaluated the labels according to the predefined performance metric, that had been published together with the training data.

### 2.3 Causality of methods

As the full test set is available to the participants from the beginning and not (as in a real online experiment) incrementally, the participants could in principle exploit the structure of the full (unlabeled) data already in advance in order to improve their label estimate even for the first trials. The organizers are aware of the problem, that this use of data is non-causal and unrealistic. Consequently it was not allowed for participants to exploit this unrealistic advantage, that they could gain compared to a BCI practitioner.

However, the distribution of test data is simplified to a large extend, if it can be provided en bloc. In order to ensure causal processing despite of this distribution method, the participants had to submit a short description of the developed data processing routines. In case of unclear causality the participants had to prove that their approach is causal by handing in the data processing routines in addition to the labels.

## 3 Data Set 1

Data set 1 *Asynchronous Motor Imagery* is provided by B. Blankertz, C. Vidaurre and K.-R. Müller from Berlin (Germany). It can be freely assessed via http://www.bbci.de/competition/iv/with the only restriction that the present article is referenced upon any publication of results.

### 3.1 Motivation

Most demonstrations of algorithms on BCI data are evaluating classification of EEG trials, i.e., segments of EEG signals of a fixed length, where each trial corresponds to a specific mental state. But in BCI applications with asynchronous feedback, e.g., cursor control, one is faced with the problem that the classifier has to be applied continuously to the incoming EEG without having cues of when the subject is switching her/his intention. This data set poses the challenge of applying a classifier to continuous EEG for which no cue information is given.

Another issue that is addressed in this data set is that the test data contains periods in which the user has *no control* intention. During those intervals the classifier is supposed to return to 0 (no affiliation to one of the target classes).

As a special feature, some of the data sets were artificially generated. The idea was to have a means for generating artificial EEG signals with specified properties that are so realistic that they can be used to evaluate and compare analysis techniques. The competition is a possibility to verify whether the applied methods perform comparably on artificial and real data. The only information provided to the competitors was that there is at least one real and at least one artificial data set, while the true distribution remained undisclosed until the submission deadline. For competition purpose, only results for the real data set(s) were considered, but results for artificial data were also reported for comparison. See the subsequent Section 4 for a detailed description of the generation of the artificial data and a comparison of the competition results obtained on real vs. artificial data.

### 3.2 Materials and subjects

These data sets were recorded exclusively for the purpose of the competition. Four healthy participants served as experimental subjects. In the whole session motor imagery was performed without feedback. For each participant *two classes* of motor imagery were selected from the three classes *left hand*, *right hand*, and *foot* (side chosen by the individual; optionally also both feet).

#### 3.2.1 Experimental paradigm

The recording was made using *BrainAmp MR plus* amplifiers (Brain Products GmbH, Munich, Germany) and a Ag/AgCl electrode cap (EASYCAP GmbH). Signals from 59 EEG positions were measured that were most densely distributed over sensorimotor areas. Signals were band-pass filtered between 0.05 and 200 Hz and then digitized at 1000 Hz with 16 bit (0.1 μV) accuracy. Also a version of the data was provided that was sub sampled at 100 Hz [first low-pass filtering the original data (Chebyshev Type II filter of order 10 with stop band ripple 50 dB down and stop band edge frequency 49 Hz) and then calculating the mean of consecutive blocks of 10 samples].

#### 3.2.2 Protocol

The session was divided into two parts: recording of training data and recording of test data. Training data were provided with complete marker information such that it could be used by the competitors for adapting the parameters of the methods/models. In contrast, the test data which was provided to the competitor only consisted of the EEG signals. The corresponding markers have been kept secret until the submission deadline and have been used to evaluate the submissions.

##### 3.2.2.1 Training data

**Figure 3:**
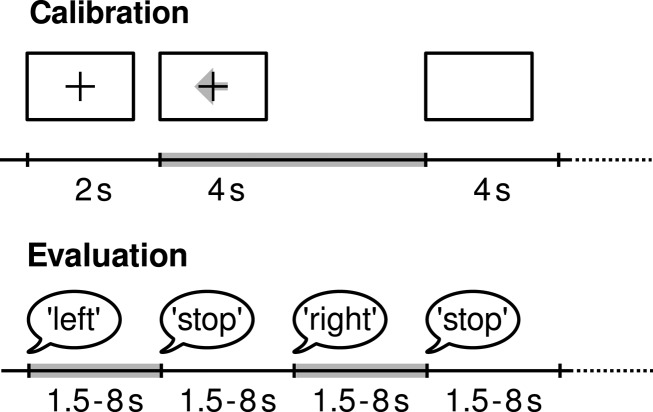
**(Data set 1 – trial structure)**. Training data was collected in the calibration runs. Arrows pointing left, right, or down have been presented as cues for imagining *left hand*, *right hand*, or *foot* movements. After a fixation cross was presented for 2 s, the directional cue was overlaid for 4 s. Then the screen was blank for 2 s. In the test runs used for evaluation, spoken words have been presented as cues.

In the first two runs, arrows pointing left, right, or down were presented as visual cues on a computer screen. Cues were displayed for a period of 4 s during which the subject was instructed to perform the cued motor imagery task. These periods were interleaved with 2 s of blank screen and 2 s with a fixation cross shown in the center of the screen. The fixation cross was superimposed on the cues, i.e., it was shown for 6 s, see Figure 3. In each run 50 trials of each of the chosen two classes have been presented, resulting in a total of 200 trials. After every 15 trials a break of 15 s was given for relaxation. Between the runs there were longer breaks of 5–15 min.

##### 3.2.2.2 Test data

Then 4 runs followed which were used for evaluating the submissions to the competitions. Here, the motor imagery tasks were cued by acoustic stimuli (words *left*, *right*, and *foot*) for periods of varying length between 1.5 and 8 s. The end of the motor imagery period was indicated by the word *stop*. Intermitting periods had also a varying duration of 1.5–8 s. The acoustical cues were soft-spoken it order to avoid that acoustically evoked potentials could be detected to segment the data into control and no-control intervals (or even to decode the cue information). In each run, 30 trials for each class have been recorded resulting in a total of 240 trials. After every 30 trials a break of 15 s was given for relaxation. Between the runs there were longer breaks of 5–15 min. Competitors were informed that the number of trials from each condition was not necessarily equal. Due to the experimental design, there were twice as much periods of no control as periods of each condition.

Additionally, we introduced a kind of non-stationarity into the test data by changing the environmental conditions. Occasionally during the runs music (2 times) or videos (2 times) have been played, or the participant was instructed to close her/his eyes (2 times). Each of those periods (during which the cue presentation was not paused) lasted about 2 min.

#### 3.3 Investigation of the data set

**Figure 4:**
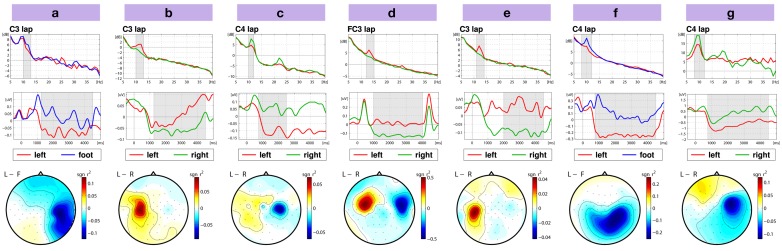
**(Data set 1 – glance at the neurophysiology)**. The first row displays the averaged spectra of the two chosen motor imagery tasks (red: left hand, green: right hand; blue: foot) in the training data. A selected subject-specific frequency band is shaded in gray. The second row shows the average amplitude envelope of that frequency band with 0 being the time point of cue presentation. The time interval which was used to calculate the spectra shown above is shaded. The bottommost row displays the (signed) *r*^2^-difference in log band-power between the individually chosen motor imagery tasks as scalp maps. Band-power was calculated in the frequency band that is shown in the topmost row and averaged across the time interval that is indicated in the middle row. Three of those seven data sets have been artificially generated, see main text.

The most stable effect of motor imagery is a modulation of the sensorimotor rhythms (SMRs), see (Pfurtscheller and da Silva, 1999). For hand motor imagery an attenuation of the SMR amplitude over the contralateral motor area is expected. The effect of foot imagery is more diverse. An attenuation of the SMR over the foot area, which is on the midline of the motor cortex could be expected, but is rarely observed and does not appear in the data set. In most of the subjects, an increase of the SMR amplitude over the hand areas is observed. This is also the case for the two participants (*a* and *f*) of this data set, who performed foot imagery. Figure 4 gives an overview, of how this effect is reflected in the competition data set. For each participant, an individual channel, time interval, and frequency band was selected to display the differential modulations of the SMRs. Class-wise averaged frequency spectra are plotted in the upper row. The second row shows the time course of band-power averaged across all trials. The bottommost row displays the difference in log band-power between the two motor imagery conditions as scalp topographies.

Data sets *c*, *d*, and *e* were artificially generated.

#### 3.4 Challenge

The submissions were evaluated in view of a one dimensional cursor control application with range from −1 to 1. The mental state of class one is used to position the cursor at −1, and the mental state of class two is used to position the cursor near 1. In the absence of those mental states (intermitting intervals) the cursor should be at position 0. Note that it is unknown to the competitors at which intervals the subject is in a defined mental state. Competitors had to submit classifier outputs for all time points. To measure the performance, the squared error with respect to the target vector – that is −1 for class one, 1 for class two, and 0 otherwise – averaged across time points was calculated. Since the mental state of the user does not abruptly change with cue appearance, time points during transient periods (1 s starting from each cue) were discarded from evaluation.

As stated above, it was declared that for competition purpose, only results for the real data sets were considered, but results for artificial data were also reported for comparison.

Additionally, participants were asked to optionally judge which of the data sets were the artificially generated ones.

#### 3.5 Outcome in brief

**Figure 5:**
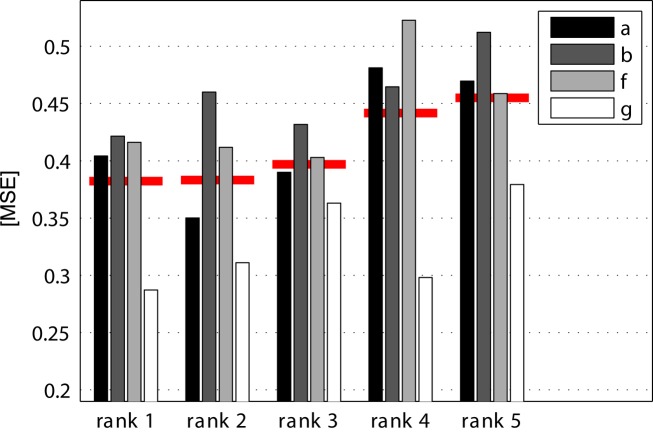
**(Data set 1 – histogram of results)**. Performance of the first five ranked submissions is shown in terms of their mean squared error (MSE) wrt. the true labels. Only results for the real (i.e., not artificially generated) data sets are shown. The mean across the four data sets is plotted as a horizontal red line. The MSE for constant prediction output of 0 are 0.507, 0.515, 0.491, 0.524 for data sets *a*, *b*, *f*, *g*, respectively.

There were 24 submissions to data set 1. The winning team is Zhang Haihong and colleagues from the Institute for Infocomm Research, Singapore. They approached the task as a three class problem with the *rest class* being the third class. For classification, CSP was combined with a filter bank. A criterion based on mutual information was used to select those features that were to be fed into a radial basis function based neural network. Using this approach, they obtained a mean squared error (MSE) of 0.382 (averaged across the four real data sets). For further details of their method see (Zhang et al., 2012). The winners are very closely followed by Dieter Devlaminck and colleagues from the University of Ghent, from the Psychiatric Institute of Guislain and from the University Hospital Ghent, who obtained an MSE of 0.383. They employed multi-class CSP with a subject-specific frequency band and a multi-class support vector machine (SVM) with ordinal regression. The results ranked 3rd to 5th have been achieved by Kai Keng Ann and colleagues (Institute for Infocomm Research, Singapore); Liu Guangquan and colleagues (Shanghai Jiao Tong University, China), and Abdul Satti and colleagues (University of Ulster). All those three competitors also used CSP as a pivotal step in combination with a filter bank (rank 3) or with a subject-specific frequency band (ranks 4 and 5). Figure 5 shows histograms of the results of those five highest ranked submissions.

**Figure 6:**
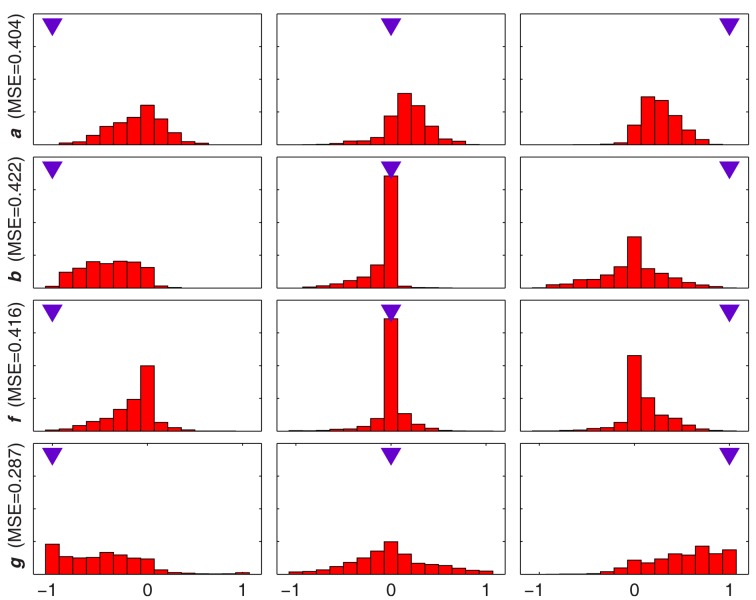
**(Data set 1 – distribution of classifier outputs)**. These (normalized) histograms display the distribution of the classifier outputs of the winning algorithm. Each row corresponds to one data set (*a*, *b*, *f*, *g*). The left column is a histogram for those time points in which the true label is −1, for the middle column it is 0 (no control), and for the right column it is 1. The true label is indicated by the blue triangle.

To assess the results, it has to be taken into account that a classifier that gives the constant output zero has an MSE of about 0.5. The exact value varies between data sets since the length of the motor imagery and no-control period was chosen randomly. Figure 6 gives a more detailed view on the performance of the winning algorithm. It shows for the four data sets (rows) normalized histograms of the classifier outputs – separately for periods of the three mental states. In the left column the true label is −1 (first motor imagery class), in the middle column the true label is 0 (no control intention), and in the right column the true label is 1 (second motor imagery condition). The value of the true label is indicated by a blue triangle in each subplot. This figure makes clear that this data set poses really a big challenge. Even for the best method among 24 submissions, the results are not very satisfying. Interestingly, the no-control state is quite well detected in the second data set (participant *b*). The overall best performance was achieved in the forth data set (participant *g*).

**Figure 7:**
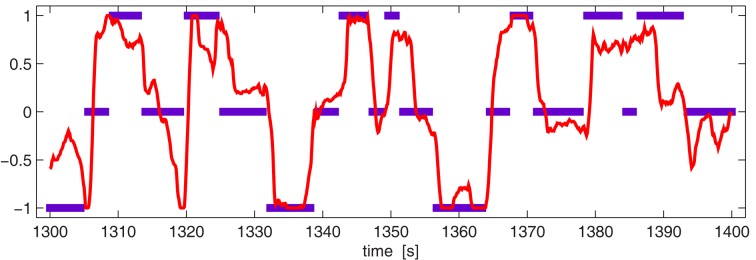
**(Data set 1 – trace of classifier outputs)**. The labels of the true mental state are displayed in blue. The red line shows the classifier outputs of the winning algorithm. This example is a selected segment of 100 s taken from data set *g* in which the classification is quite successful. The MSE in the shown segment is 0.171.

Figure 7 gives a better intuition of how well the obtained control actually is. It shows for a selected segment of 100 s the true mental state (blue bars) and the classifier output of the winning algorithm (red line).

A guess on the question which data sets were artificially generated was submitted by 16 out of 23 competitors. The correct categorization was revealed by two competitors (Astrid Zeman and Manuel Moebius), 8 more competitors revealed 2 of the 3 artificial subjects, but one of those also considered one real data set as artificial.

## 4 Data Set 1 (Artificially Generated)

The subset of Data set 1 that was artificially generated is provided by C. Vidaurre and G. Nolte from Berlin (Germany). It can be freely assessed via http://www.bbci.de/competition/iv/ with the only restriction that the present article is referenced upon any publication of results.

### 4.1 Motivation

The BCI competition IV included an original ingredient compared to past events: part of the data sets of the BBCI group were artificially generated. The motivation of this work was to check whether or not EEG data can be created to have specific properties in order to test new machine learning methods. If this was the case, the algorithms applied to both, synthetic and real EEG, would produce comparable results. To test this hypothesis we analyzed the ranking of the participants and the performance of the methods in real and synthetic EEG. Brain data like EEG is often noisy and its variables cannot be controlled easily. Synthetically generated data may overcome these difficulties, and besides it is easy and cheap to produce.

### 4.2 Material

In the following, the single components of (artificial) EEG are described separately. We start with the generation of artificial EEG noise, which we divided into background noise and baseline drifts. Then we describe the generation of the μ (and β as a first harmonic of μ) rhythm and its desynchronization (ERD) due to the onset of motor imagery tasks. After that, we describe the artifacts that have been added to the data (eye blinks and eye movements) to include some more realistic noise in our signals. Our synthetic EEG is computed as a superposition of potentials from these three different systems (ongoing background noise, task dependent rhythmic activity generated on the motor cortex and eye related noise, eye blinks, and eye movements) which have qualitatively different statistical and spatial properties.

#### 4.2.1 Background noise

Background noise in EEG can reasonably be assumed to be Gaussian distributed. The spatial and temporal characteristics, however, are in general too complex to be adequately modeled using a simple parametric model (Huizenga et al., 2002; Bijma et al., 2003; Freeman, 2004a,b, 2005, 2006). To solve this difficulty, we first estimated the cross-spectrum from real EEG data in *eyes open* and *eyes closed* conditions and then generated an arbitrary amount of data according to the estimated (complex) cross-spectral matrix in the following way:

Let *x_i_*(*f* ) be the Fourier transform of simulated white Gaussian noise for *N* data points for channel *i* with *i* = 1…*M*. Since typically (and in the case of our data) the estimated cross-spectrum *C*(*f_i_*) at discrete frequencies *f_i_* is based on averages of relatively short time windows (duration: 1 s) and *N* denotes the length of the complete data set, the frequency resolution of the measured cross-spectra is much lower than the frequency resolution of *x_i_*(*f*). We estimate *C*(*f *) as a linear interpolation:

(1) $$C\left(f\right)\equiv =\frac{f-f_{1}}{f_{2}-f_{1}}C\left(f_{1}\right)+\frac{f_{2}-f}{f_{2}-f_{1}}C\left(f_{2}\right)$$

with *f*_1_ (*f*_2_) being the largest (smallest) value of the set (*f_i_*) lower (higher) than *f*. Then we scale **x** ≡ (**x**_1_(**f**), **x_2_**(**f**),…,**x_M_**(**f**))**^T^** with A(*f *) defined by the decomposition^1^ *C*(*f *) = *A*(*f *)*A*(*f *)^†^ with ^†^ denoting transpose and complex conjugation:

(2) y(f)=A(f)x(f)

**Figure 8:**
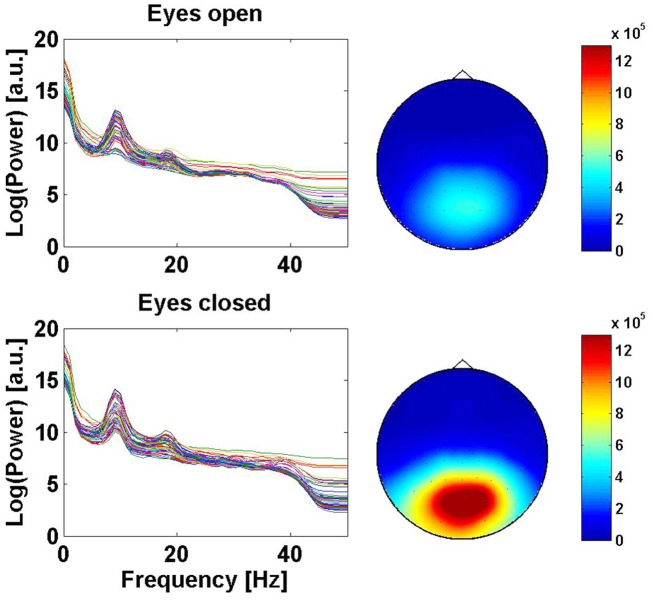
**(Data Set 1 – artificial)**. Left: spectra of the signal at all channel locations for the two conditions, eyes open and eyes closed. Right: scalp plot of the signal power at 10 Hz for the two conditions (eyes open and closed). The actual noise of the artificial data varied linearly in time between both conditions, depending of the task of the BCI user.

Finally, the simulated noise data in the channel *i* is calculated as the inverse Fourier transform of *y_i_*(*f *). The resulting background noise was a superposition of different amounts of each type of noise, depending on the condition. Figure 8 depicts noise in conditions *eyes open* and *eyes closed* at the 10-Hz frequency. One can observe that the power at this frequency is varying in the occipital region, as expected in real EEG data.

#### 4.2.2 Baseline drifts

**Figure 9:**
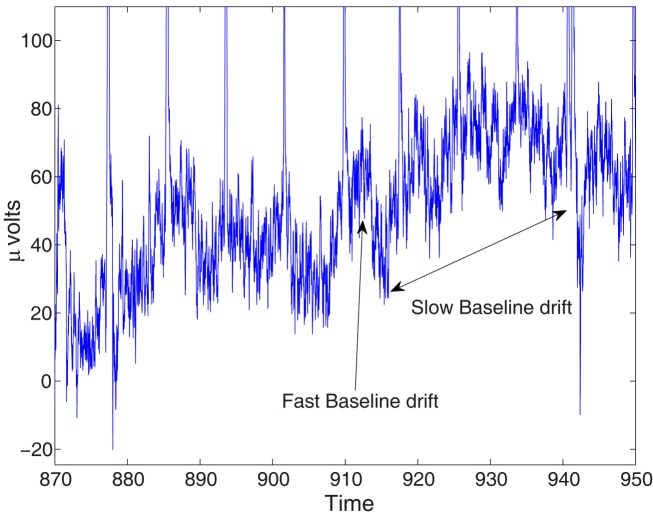
**(Data Set 1 – artificial)**. Example of fast and slow baseline drifts that are observable in unfiltered BCI competition IV data. The figure depicts the time course of the amplitude of the EEG in one channel.

Baseline drifts are typically observable in unfiltered electroencephalographic signals (cf. Simons et al., 1981; Henninghausen et al., 1993) and this is also the case for the BCI-competition data. After analyzing the real “raw” EEG of the competition, we observed both, relatively fast and slow drifts of the signal (shown in Figure 9) and accordingly created two types of artificial drifts. These drifts were generated using the cross-spectrum of the background noise, but simulating a higher sampling frequency, which had the effect of producing a slower signal (noise) than the background itself. The selected frequencies for this computation were 150 and 300 kHz, respectively (the original sampling frequency was 1 kHz).

#### 4.2.3 Event-related desynchronization

**Figure 10:**
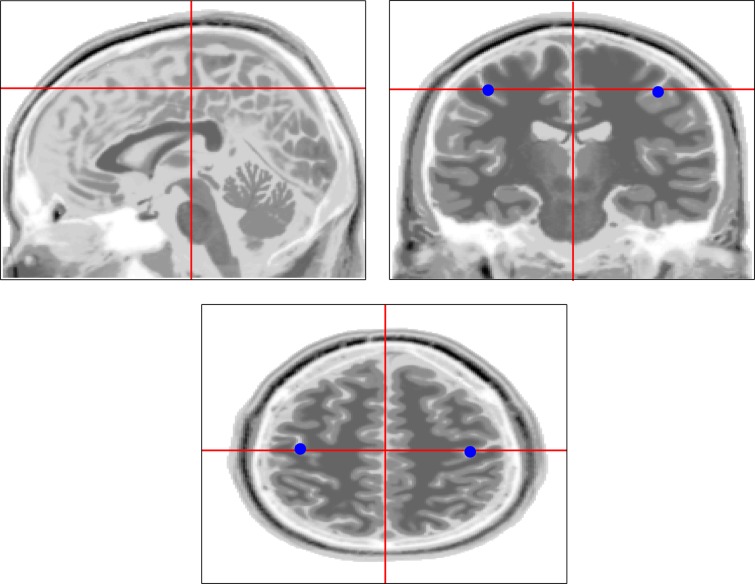
**(Data Set 1 – artificial)**. Location and direction of selected dipoles in the head.

**Figure 11:**
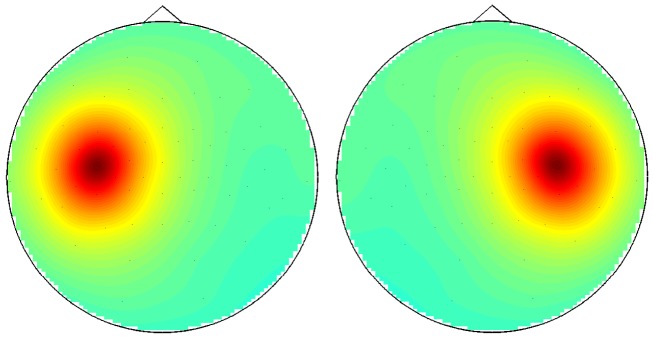
**(Data Set 1 – artificial)**. Power topographies at the μ rhythm frequency generated by the dipoles.

Forward calculation For the generation of ERD we assumed fixed spatial patterns calculated as potential maps from dipolar sources within left and right motor areas (Geselowitz, 1967). Again, we assumed that the data is Gaussian distributed. The frequency content, however, was restricted to a single frequency (chosen to be 12 Hz) with a width δ*f* = 1 Hz. We assumed that the generators of this rhythm were radial dipoles with the origins to be 3 cm below electrodes C3 and C4 for the left and right side activity, respectively (see Figures 10 and 11). The directions “radial” and also “below” were chosen according to the surface normals at electrodes C3 and C4. For the forward calculation we assumed a realistic volume conductor consisting of three shells (scalp, skull, brain) with conductivity ratios 1:0.02:1. The Maxwell equations were solved using an analytic expansion of the EEG lead field (Nolte and Dassios, 2005).

Both left and right rhythmic activity was present in all conditions. However, during left hand movements the right side rhythmic activity was reduced by at least 50% (it changed slightly for each data set) and vice versa.

#### 4.2.4 Harmonic oscillations in the beta band

**Figure 12:**
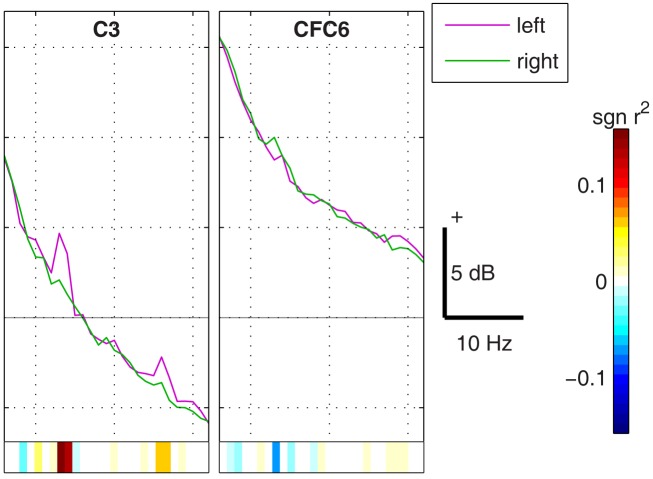
**(Data Set 1 – artificial)**. Spectra of the EEG signal in two discriminative channels. The discriminability between the classes is shown at the bottom of each spectrum. This Figure illustrates an example of asymmetry of the μ rhythm peak in each hemisphere. Also the harmonic of the μ rhythm is observable in the beta band.

A harmonic component of the subject-specific μ rhythm can often be observed in the β band (Huber et al., 1971; Pfurtscheller, 1981; Pfurtscheller et al., 1996; Pfurtscheller and Lopes da Silva, 1999; Carlqvist et al., 2004; Nikulin et al., 2007). In our data sets we have included such a harmonic component with different levels of amplitude in relation to the μ rhythm, varying from 15 to 1% (see Figure 12).

#### 4.2.5 Asymmetry in the amplitude of the rhythms

Typically, one can observe some asymmetry in the strength of the desynchronization in each of the hemispheres (McFarland et al., 2000; Mazaheri and Jensen, 2008; Nikulin et al., 2010). In a pair of data sets and in order to create a more realistic EEG we added this asymmetry in the rhythms that we generated.

#### 4.2.6 Generation of artifacts

**Figure 13:**
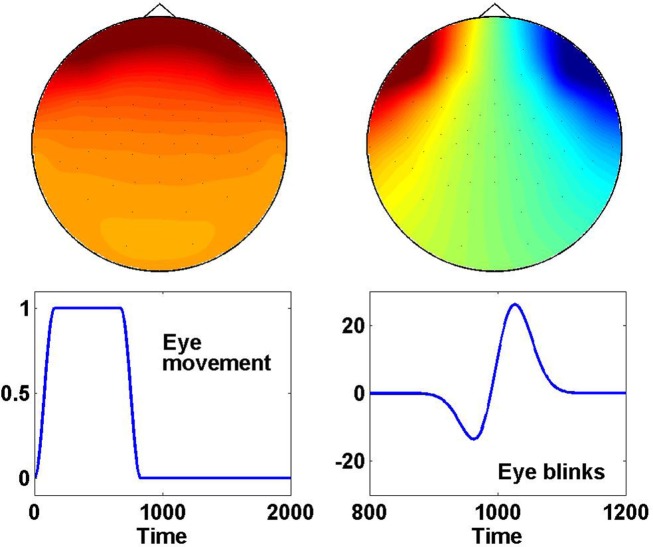
**(Data Set 1 – artificial)**. Top row: scalp plots of one eye movement and one eye blink generated for the synthetic EEG data sets of the BCI competition IV. Bottom row: corresponding time course of eye movements and blinks.

Both for eye blinks and eye movements we assumed the generators to be current dipoles placed within the eyes. The dipoles in the left and right eye were activated simultaneously in a randomly chosen superposition of a vertical and horizontal direction. The potentials due to vertical dipoles were on average 10 times stronger than the ones from horizontal direction. The topographies of vertical and horizontal dipoles are shown in the upper panels of Figure 13.

While the spatial patterns were (on average) identical for eye movement and eye blinks, the time courses were chosen differently. Time courses of eye movements were modeled as constants with continuous on- and offsets as shown in the lower left panel of Figure 13. The duration of the constant was set randomly between 0 and 2 s.

The time course of eye blinks was chosen as

(3) $$x\left(t\right)=\left(t+\xi \right)\exp\left(-\frac{t^{2}}{2\sigma_{t}^{2}}\right)$$

with a width set to σ*_t_* = 31 ms according to real eye blinks and with *xi* being a Gaussian distributed random variable with standard deviation equal to 20 ms. An example time course is given in the lower right panel of Figure 13.

#### 4.2.7 Combining the ingredients

For each data set, the final EEG was generated by the linear combination of each element (background noise, baseline drifts, ERD, and eye artifacts). The background noise was a superposition of the cross-spectra in the conditions *eyes open* and *eyes closed*. The amount of each type of cross-spectrum depended on the “environmental” conditions in which the virtual user was supposed to be immersed: visual load (large amount of *eyes open* condition and small amount of *eyes closed* condition), auditive load (small amount of *eyes open* condition and larger amount of *eyes closed* condition).

The ERD frequency was randomly chosen between 10 and 12 Hz for each user and a harmonic in the beta band (by doubling the μ rhythm frequency) was added as well. The position of the dipoles generating the oscillatory activity could vary slightly and randomly for each user. As already described, we also allowed asymmetry of the μ rhythm amplitude in each hemisphere.

Then the eye movements and eye blinks were as well superimposed to the signal. One random time course was generated for each of the data sets. Finally, the baseline drifts were added to the total.

For the calculation, each element (ERD, background noise, etc.) was normalized by its trace and the coefficient multiplying each of them was manually selected, by computing the expected performance using baseline methods (frequency band and time interval subject-selected, then CSP computed using training data and applied to test data). For more information please refer to (Blankertz et al., 2008b).

### 4.3 Challenge

As it was not revealed which of the data sets were real and which were artificially generated. The challenge and evaluation criterion was identical, see Section 3.4.

### 4.4 Outcome in brief

A comparison of the similarity of real and synthetic data was performed based on the result ranking (available via http://bbci.de/competition/iv/results/). First, we analyzed the position of the participants in the ranking. We calculated the correlation coefficient of the participants’ positions in both of the data sets and obtained a result of 0.89, meaning that the position of a participant in both rankings was highly correlated: a good rank in the real data analysis yielded a good rank in the artificial data analysis and vice versa. Also, we analyzed the performance of the participants in the same way. We obtained a correlation coefficient of 0.93, meaning that the performance of a participant in both data sets was very similar. The linear fitting had a slight positive bias (0.02), which shows that the performance measurement (mean squared error) was slightly higher for the synthetic EEG (these data sets were a bit noisier than the real ones).

Summarizing, those algorithms doing well in the real data sets also performed higher in the artificial data and vice versa.

### 4.5 Discussion

In this section we gave a description of the generation of synthetic EEG. We have described all its components, documented our decisions, and detailed the calculation of each element.

We emphasize that more sophisticated EEG forward models would include CSF as a fourth layer and that new research indicates that the chosen conductivity ratio (1:50) might be too high. While the simulation could be improved, almost all BCI methods work entirely in sensor space and the exact details of the topographies will hardly affect the results of the BCI task.

**Figure 14:**
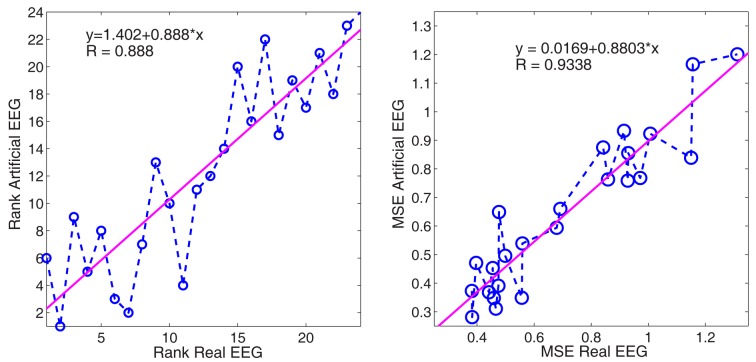
**(Data Set 1 – artificial)**. Linear regression of the rank position (left) and performance of the method (right). The x-axis corresponds to the results submitted for the real EEG data sets, whereas y-axis corresponds to those of the synthetic EEG.

We have analyzed the results of the BCI competition IV and shown the high correlation between the ranking and the performance measure in the real and artificial EEG in Figure 14. In this specific context, the creation of synthetic EEG data sets has proven to be useful.

Artificially generated EEG can be generated in large amounts. Using it not only avoids performing real recordings, it can also be fine-tuned, e.g., to contain a controlled amount of certain artifacts. Both characteristics are beneficial for an initial performance evaluation of new algorithmic methods.

Although this was our first try to generate artificial data and the methods can be further developed, we have shown a way to create data under controlled conditions, in order to test new methods before performing actual experiments and this way boosting the probability of success of new analysis methods in neuroscience.

In the future we will work on the improvement of the artifact generation methods and develop an automatic way to combine all the components of our synthetic electroencephalogram. Additionally, more tests should be done with the artificially generated data, to assure that the correlation between real and synthetic EEG is as high as shown in this report.

## 5 Data Set 2a

Data set 2a *Continuous Multi-class Motor Imagery* is provided by C. Brunner, R. Leeb, G. R. Müller-Putz, G. Pfurtscheller, and A. Schlögl from Graz (Austria). It can be freely assessed via http://www.bbci.de/competition/iv/ with the only restriction that the present article is referenced upon any publication of results.

### 5.1 Motivation

This data set challenges the session-to-session transfer of a three class motor imagery task. Compared to other synchronous motor imagery data sets, a continuous estimation of motor imagery class labels is required. This represents a realistic setting for an online control of a continuous output parameter.

### 5.2 Materials and subjects

This data set comprises electroencephalographic (EEG) data from 9 subjects.

#### 5.2.1 Experimental paradigm

The cue-based BCI paradigm consisted of four different motor imagery tasks, namely the imagination of movement of the left hand (class 1), right hand (class 2), both feet (class 3), and tongue (class 4). Two sessions on different days were recorded for each subject. Each session is comprised of 6 runs separated by short breaks. One run consists of 48 trials (12 for each of the four possible classes), yielding a total of 288 trials per session.

#### 5.2.2 Protocol

**Figure 15:**
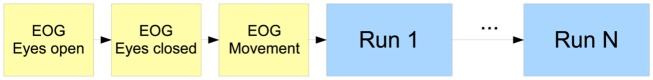
**(Data Set 2a)**. Timing scheme of one session.

At the beginning of each session, we recorded approximately 5 min of EEG data to estimate the EOG influence. This recording was divided into 3 blocks: (1) 2 min with eyes open (looking at a fixation cross on the screen), (2) 1 min with eyes closed, and (3) 1 min with eye movements. The timing scheme of one session is illustrated in Figure 15. Note that due to technical problems, the EOG block is shorter for subject A04T and contains only the eye movement condition (see Table A1 in Appendix for a list of all subjects).

**Figure 16:**
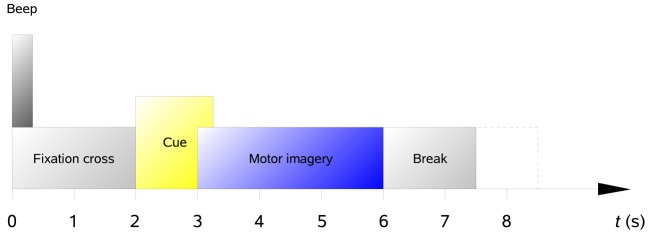
**(Data Set 2a)**. Timing scheme of the paradigm.

All subjects were sitting in a comfortable armchair in front of a computer screen. At the beginning of a trial (*t* = 0 s), a fixation cross appeared on the black screen. In addition, a short acoustic warning tone was presented. After 2 s (*t* = 2 s), a cue in the form of an arrow pointing either to the left, right, down, or up (corresponding to one of the four classes left hand, right hand, foot, or tongue) appeared and stayed on the screen for 1.25 s. This prompted the subjects to perform the desired motor imagery task. No feedback was provided. The subjects were instructed to carry out the motor imagery task until the fixation cross disappeared from the screen at *t* = 6 s. A short break with a black screen followed. The paradigm is illustrated in Figure 16.

### 5.3 Data format

**Figure 17:**
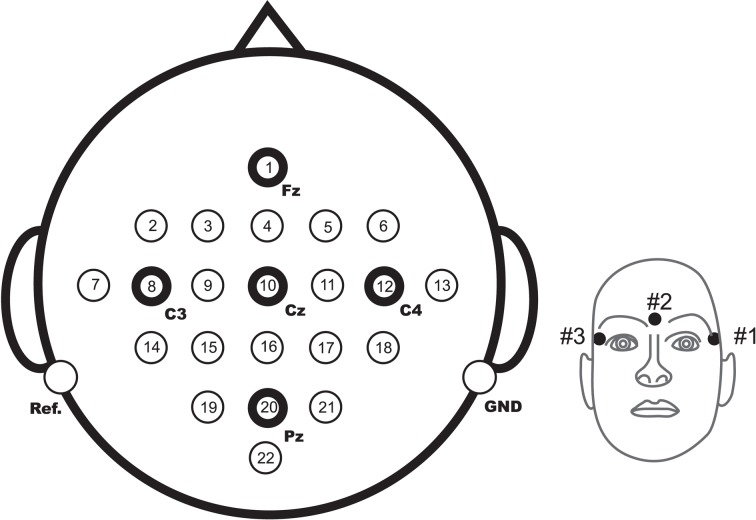
**(Data Set 2a)**. Left: electrode montage corresponding to the international 10–20 system. Right: electrode montage of the three monopolar EOG channels.

Twenty-two Ag/AgCl electrodes (with inter-electrode distances of 3.5 cm) were used to record the EEG; the montage is shown in Figure 17, left. All signals were recorded monopolarly with the left mastoid serving as reference and the right mastoid as ground. The signals were sampled with 250 Hz and bandpass filtered between 0.5 and 100 Hz. The sensitivity of the amplifier was set to 100 μV. An additional 50 Hz notch filter was enabled to suppress line noise.

In addition to the 22 EEG channels, 3 monopolar EOG channels were recorded and also sampled with 250 Hz (see Figure 17, right). They were bandpass filtered between 0.5 and 100 Hz (with the 50-Hz notch filter enabled), and the sensitivity of the amplifier was set to 1 mV. The EOG channels are provided for the subsequent application of artifact processing methods (Fatourechi et al., 2007) and must not be used for classification.

A visual inspection of all data sets was carried out by an expert and trials containing artifacts were marked. Eight out of the total of nine data sets were analyzed in Naeem et al. (2006) and Brunner et al. (2007, 2011).

All data sets are stored in the general data format for biomedical signals (GDF), one file per subject and session. However, only one session contains the class labels for all trials, whereas the other sessions are used to test the classifier and hence to evaluate the performance. For details on the data set, the GDF files contained, markers and functions provided for loading and evaluation, please see Section A.1 in Appendix.

### 5.4 Challenge

Participants were asked to provide a continuous classification output for each sample in the form of class labels (1–4), including labeled trials and trials marked as artifact. A confusion matrix was then built from all artifact-free trials for each time point. From these confusion matrices, the time course of the accuracy as well as the kappa coefficient was obtained (Schlögl et al., 2007b). The chance level was at κ = 0. The algorithm used for this evaluation was provided in BioSig. The algorithm achieving the largest kappa value was declared the winner.

Due to the fact that the test data sets were not distributed until the end of the competition, software had to be submitted. It had to be capable to process EEG data files of the same format as used for all training sets^2^) and produce the aforementioned class label vector.

Since three EOG channels were provided, the software was required to remove EOG artifacts before the subsequent data processing using artifact removal techniques such as high pass filtering or linear regression (Schlögl et al., 2007a). The use of other correction methods was possible, but it was requested that artifacts had no influence on the classification results.

All algorithms had to be causal, meaning that the classification output at time *k* was allowed only to depend on the current and past samples *x_k_,x_k−1_,…,x*_0_. In order to check whether the causality criterion and the artifact processing requirements were fulfilled, all submissions had to be open source, including all additional libraries, compilers, programming languages, and so on (for example, Octave/FreeMat, C++, Python, etc.). Note that submissions could also be written in the closed-source development environment MATLAB as long as the code was executable in Octave. Similarly, C++ programs could be written and compiled with a Microsoft or Intel compiler, but the code had to compile also with g++.

### 5.5 Outcome in brief

There were five submissions for this data set (see Table 3). All of them used CSP features. The winning algorithm was submitted by K. K. Ang, Z. Y. Chin, C. Wang, C. Guan, H. Zhang, K. S. Phua, B. Hamadicharef, and K. P. Tee from the Institute for Infocomm Research, Agency for Science, Technology and Research Singapore. Details of their approach are described in Ang et al. (2012). The performance measure kappa was 0.57 averaged over all nine subjects. The other four submissions attained kappa values of 0.52, 0.31, 0.30, and 0.29 and thus were well above chance level of κ = 0. The winning algorithm performed best in seven out of nine subjects; in two subjects, the algorithm that overall ranked second best reached even slightly higher kappa values.

**Table 3:** **(Data Set 2a)**. Contributions with final result (kappa).

Contributor	Kappa	Lab
K. K. Ang	0.57	Institute for Infocomm Research, Agency for Science, Technology and Research Singapore
L. Guangquan	0.52	School of Mechanical Engineering, Shanghai Jiao Tong University, China
W. Song	0.31	College of Inf. Science and Techn., Beijing Normal University, China and National Key Lab. or Cog. Neurosc. and Learning, Beijing Normal Univ., China
D. Coyle	0.30	Intelligent Systems Research Centre, School of Computing and Intell. Systems, Faculty of Computing and Eng., Magee Campus, University of Ulster, UK
J. Wu	0.29	National Key Lab. for Cogn. Neurosc. and Learning, Beijing Normal Univ., China and College of Inf. Science and Techn., Beijing Normal University, China

The winning algorithm requires MATLAB, but also runs on Octave. It uses the BioSig toolbox to load the data. The algorithm is based on the filter bank common spatial pattern (FBCSP) variant (Ang et al., 2008). It was extended to the multi-class case with one-versus-the-rest classifiers. First, artifacts were removed by bandpass filters. Each classifier selected discriminative CSP features using the Mutual Information Best Individual Features (MIBIF4) algorithm (Ang and Quek, 2006) before Naive Bayes Parzen Window classifiers (Ang and Quek, 2006) were used. The classifier with the highest probability yielded the overall classification result. Due to the computationally intensive algorithms, classification was performed every ten samples (in combination with a zero-order hold for the samples in between). As the algorithm used 2 s of EEG data, the classification output was delayed by 2 s.

### 5.6 Discussion

All five submissions yielded results well above chance level. As a side note, four contributions were submitted by Asian-Pacific groups. As already mentioned above, all contributions used CSP features.

There were two major challenges in this data set. First, the contamination with eye movement artifacts could affect classification accuracy; therefore we provided additional EOG channels. Second, the classifiers trained on the training sessions should generalize on unseen data recorded on a different day. The winning algorithm addressed the first issue with a simple bandpass filter. Obviously, the method is stable because it yielded good results on the test data. However, the classification output is delayed by 2 s, which could be a problem in online BCIs that incorporate real-time feedback.

## 6 Data Set 2b

Data set 2b *Session-to-Session Transfer of a Motor Imagery BCI under Presence of Eye Artifacts* is provided by R. Leeb, C. Brunner, G. R. Müller-Putz, and G. Pfurtscheller from Graz (Austria). It can be freely assessed via http://www.bbci.de/competition/iv/ with the only restriction that the present article is referenced upon any publication of results.

### 6.1 Motivation

This data set focuses on the classification of electroencephalogram (EEG) signals affected by eye movement artifacts. Furthermore the session-to-session transfer of the algorithms has to be taken in consideration, because all training and test data sets are recorded on five different days.

The data set 2b contains the electroencephalogram (EEG) and electrooculogram (EOG) activity of nine subjects. Technically speaking, each data set consists of single-trials of spontaneous brain activity during motor imagery, one part labeled (training data) and another part unlabeled (test data), and a performance measure. The goal is to infer labels (or their probabilities) for the test data sets from training data that maximize the performance measure for the true (but to the competitors unknown) labels of the test data (this information is now, after the competition, available as well).

### 6.2 Materials and subjects

This data set consists of EEG data from 9 subjects of a study published in Leeb et al. (2007). The subjects were right-handed, had normal or corrected-to-normal vision and were paid for participating in the experiments. All volunteers were sitting in an armchair, watching a flat screen monitor placed approximately 1 m away at eye level. For each subject 5 sessions are provided, whereby the first two sessions contain training data without feedback (screening), and the last three sessions were recorded with feedback.

#### 6.2.1 Experimental paradigm

**Figure 18:**
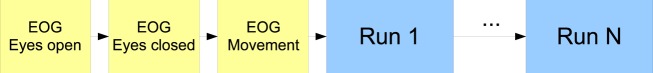
**(Data Set 2b)**. Timing scheme of one session (for screening and feedback sessions).

Each session consists of several runs, illustrated in Figure 18. At the beginning of each session, a recording of approximately 5 min was performed to estimate the EOG influence. The recording was divided into 3 blocks: (1) 2 min with eyes open (looking at a fixation cross on the screen), (2) 1 min with eyes closed, and (3) 1 min with eye movements. The artifact block was divided into four sections (15 s artifacts with 5 s resting in between) and the subjects were instructed with a text on the monitor to perform either eye blinking, rolling, up-down, or left-right movements. At the beginning and at the end of each task a low and high warning tone were presented, respectively. Note that due to technical problems no EOG block is available in session B0102T and B0504E (see Table A3 in Appendix for a list of all subjects).

#### 6.2.2 Protocol

Three bipolar recordings (C3, Cz, and C4) were recorded with a sampling frequency of 250 Hz. The recordings had a dynamic range of ±100 μV for the screening and ±50 μV for the feedback sessions. They were bandpass filtered between 0.5 and 100 Hz, and a notch filter at 50 Hz was enabled. The placement of the three bipolar recordings (large or small distances, more anterior or posterior) were slightly different for each subject (for more details see Leeb et al., 2007). The electrode position Fz served as EEG ground.

**Figure 19:**
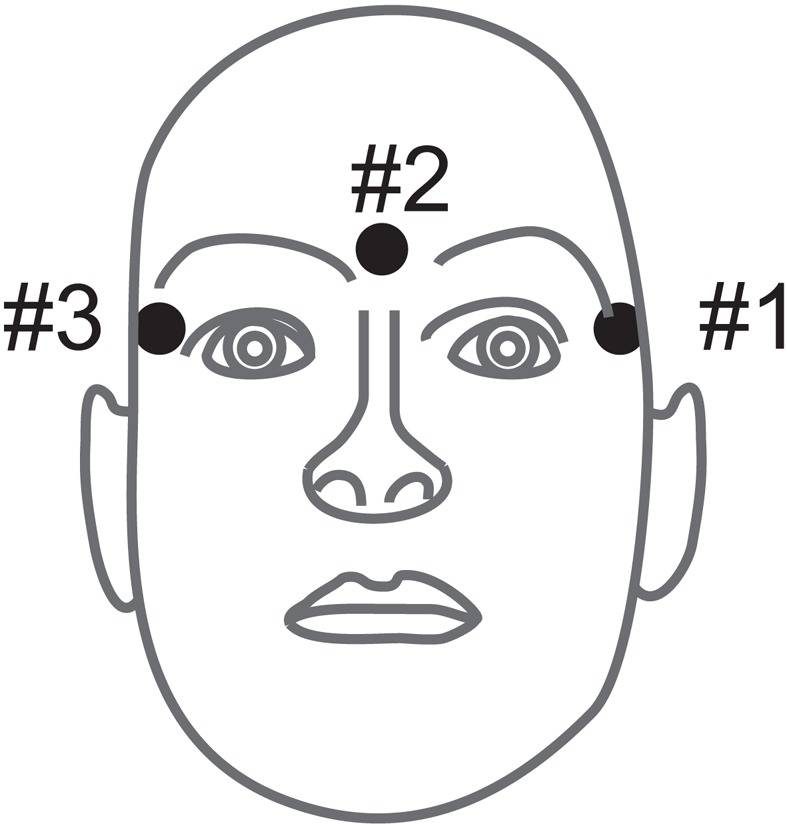
**(Data Set 2b)**. Electrode montage of the three monopolar EOG channels.

In addition to the EEG channels, the electrooculogram (EOG) was recorded with three monopolar electrodes (see Figure 19, left mastoid serving as reference) using the same amplifier settings, but with a dynamic range of ±1 mV. The EOG channels are provided for the subsequent application of artifact processing methods (Fatourechi et al., 2007) and must not be used for classification.

**Figure 20:**
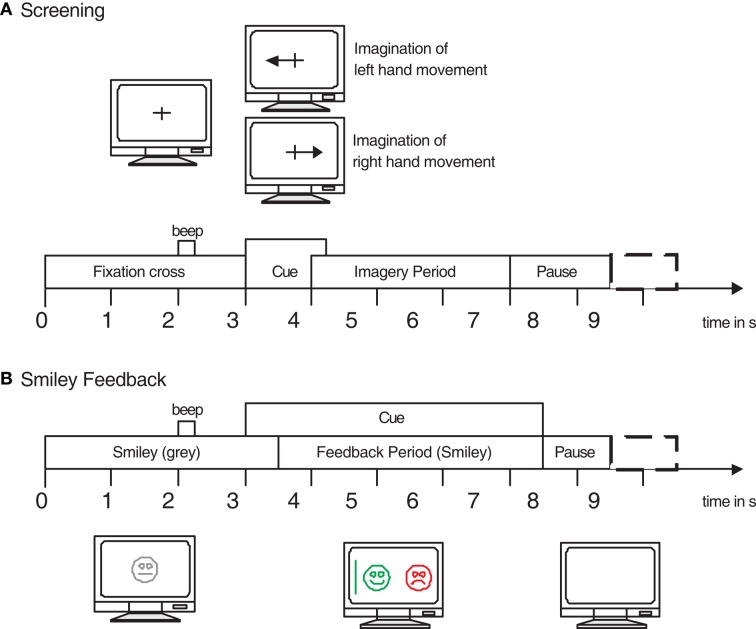
**(Data Set 2b)**. Timing scheme of the paradigm. **(A)** The first two sessions (01T, 02T) contain training data without feedback, and **(B)** the last three sessions (03T, 04E, 05E) with smiley feedback.

The cue-based screening paradigm (see Figure 20A) consisted of two classes, namely the motor imagery (MI) of left hand (class 1) and right hand (class 2). Each subject participated in two screening sessions without feedback recorded on two different days within 2 weeks. Each session consisted of six runs with ten trials each and two classes of imagery. This resulted in 20 trials per run and 120 trials per session. Data of 120 repetitions of each MI class were available for each person in total. Prior to the first motor imagery training the subject executed and imagined different movements for each body part and selected the one which they could imagine best (e. g., squeezing a ball or pulling a brake).

Each trial started with a fixation cross and an additional short acoustic warning tone (1 kHz, 70 ms). Some seconds later a visual cue (an arrow pointing either to the left or right, according to the requested class) was presented for 1.25 s. Afterward the subjects had to imagine the corresponding hand movement over a period of 4 s. Each trial was followed by a short break of at least 1.5 s. A randomized time of up to 1 s was added to the break to avoid adaptation.

For the three online feedback sessions four runs with smiley feedback were recorded (see Figure 20B), whereby each run consisted of twenty trials for each type of motor imagery. At the beginning of each trial (second 0) the feedback (a gray smiley) was centered on the screen. At second 2, a short warning beep (1 kHz, 70 ms) was given. The cue was presented from seconds 3 to 7.5. Depending on the cue, the subjects were required to move the smiley toward the left or right side by imagining left or right hand movements, respectively. During the feedback period the smiley changed to green when moved in the correct direction, otherwise it became red. The distance of the smiley from the origin was set according to the integrated classification output over the past 2 s (more details see Leeb et al., 2007). Furthermore, the classifier output was also mapped to the curvature of the mouth causing the smiley to be happy (corners of the mouth upwards) or sad (corners of the mouth downward). At second 7.5 the screen went blank and a random interval between 1.0 and 2.0 s was added to the trial. The subject was instructed to keep the smiley on the correct side for as long as possible and therefore to perform the MI as long as possible.

### 6.3 Data format

All data sets are stored in the general data format for biomedical signals (GDF), one file per subject and session. However, only the first three sessions contain the class labels for all trials, whereas the remaining two sessions are used to test the classifier and hence to evaluate the performance. For details on the data set, the GDF files contained, markers and functions provided for loading and evaluation, please see Section A.2 in Appendix.

### 6.4 Challenge

Participants were asked to provide a continuous classification output for each sample in the form of class labels (1, 2), including labeled trials and trials marked as artifacts. A confusion matrix was then built based on artifact-free trials only and for each time point. From these confusion matrices, the time course of the accuracy as well as the kappa coefficient was obtained (Schlögl et al., 2007b), which had a chance level of κ = 0. The algorithm used for this evaluation was provided in BioSig. The winner was the algorithm with the largest kappa value.

Due to the fact that the evaluation data sets were not distributed until the end of the competition, software had to be submitted. It had to be capable to process EEG data files of the same format as used for all training sets^3^) and produce the aforementioned class label vector.

Since three EOG channels were provided, the software was required to remove EOG artifacts before the subsequent data processing using artifact removal techniques such as high pass filtering or linear regression (Schlögl et al., 2007a). The use of other correction methods was possible, but it was requested that artifacts had no influence on the classification results.

All algorithms were required to be causal, meaning that the classification output at time *k* may only depend on the current and past samples *x_k_,x_k−1_,…,x*_0_. In order to check whether the causality criterion and the artifact processing requirements were fulfilled, all submissions had to be submitted as open source, including all additional libraries, compilers, programming languages, and so on (for example, Octave/FreeMat, C++, Python, etc.). Note that submissions could also be written in the closed-source development environment MATLAB as long as the code was executable in Octave. Similarly, C++ programs could be written and compiled with a Microsoft or Intel compiler, but the code had to compile also with g++.

### 6.5 Submissions and algorithms

Six groups submitted their participation for this data set. The given list is in winning order and this ID will be used further on:

ID-1: Zheng Yang Chin, Kai Keng Ang, Chuanchu Wang, Cuntai Guan, Haihong Zhang, Kok Soon Phua, Brahim Hamadicharef, and Keng Peng Tee from the Institute for Infocomm Research, Agency for Science, Technology, and Research in Singapore.
ID-2: Huang Gan, Liu Guangquan, and Zhu Xiangyang from the School of Mechanical Engineering, Shanghai Jiao Tong University in China.
ID-3: Damien Coyle, Abdul Satti, and Martin McGinnity from the Intelligent Systems Research Centre, School of Computing and Intelligent Systems, Faculty of Computing and Engineering, Magee Campus, University of Ulster in the United Kingdom.
ID-4: Shaun Lodder and Johan du Preez from the E&E Engineering, University of Stellenbosch in South Africa.
ID-5: Jaime Fernando Delgado Saa from the Robótica y Sistemas Inteligentes, Universidad del Norte in Colombia.
ID-6: Yang Ping, Xu Lei, and Yao Dezhong from the Perception-Motor Interaction Lab, School of Life Science and Technology, University of Electronic Science and Technology in China.

For each method the applied preprocessing, feature extraction, and classification steps are briefly given.

Methods participant ID-1: They authors removed the EOG with a bandpass filter and extracted their features via a Filter Bank CSP (FBCSP) using mutual information rough set reduction (MIRSR). Classification of selected CSP features was performed using the Naïve Bayes Parzen Window classifier. A more detailed explanation of the winning algorithm is given in a separate paper (Ang et al., 2012).
Methods participant ID-2: The EEG was bandpass filtered in different frequency bands and the EOG artifacts were removed afterward. Common spatial subspace decomposition (CSSD) were extracted from the preprocessed signals with optimized window sizes and a LDA discriminate function was made for each time point.
Methods participant ID-3: CSP on spectrally filtered neural time series prediction preprocessing (NTSPP) signals was applied to all signals all subjects using the self-organizing fuzzy neural network (SOFNN). Furthermore the log variance of each filtered channel was calculated with a 1-s sliding window. The best classifier among 3 variants of LDA and 2 variants of SVM was chosen for each subject individually.
Methods participant ID-4: Wavelet packet transform was applied only on electrodes C3 and C4 (Cz was ignored). Selected frequency bands were extracted and concatenated to form a multidimensional vector and classified with LDA.
Methods participant ID-5: EOG was removed with linear regression and the signals high pass filtered with 4 Hz. The algorithm used spectral features in the mu and beta bands (from electrode C3 and C4) as inputs for a neural network classifier.
Methods participant ID-6: EOG was removed with linear regression. Band-power features in 75 frequency bands for each channel were extracted and selected with recursive feature elimination (RFE). The remaining 6 features were classified with a Bayesian LDA.

### 6.6 Results

In total six submissions were received and most were of high quality. As defined above in section evaluation the kappa value was chosen as the performance measure. Remember, the expected kappa value, if classification is made by chance, is 0. In Table 4 the first column shows the average kappa across all subjects, columns 2–10 show the results for the individual subjects. Four submissions achieved a mean kappa of more than 0.4 on the test set. Furthermore the two best approaches (ID-1 and ID-2; Ang et al., 2012) achieved nearly similar results (mean of 0.60 and 0.58). Actually approach ID-2 could achieve the best single subject performances for 4 subjects and ID-1 “just” for 3 subjects, but was always very close to the best ones on a single subject level. Only subject 2 caused troubles to these algorithms. Interestingly is that approach ID-4 achieved incredible good results here compared to the other approaches. Generally the data from subject 8 and subject 4 could be identified best, whereby subjects 3, 2, and 1 were challenging. These findings are consistent over all approaches, if the standard deviation over the approaches is taken into consideration.

**Table 4:** **(Data Set 2b)**. Detailed results from the BCI competition IV.

Part. ID	Mean	Subject								
		1	2	3	4	5	6	7	8	9
ID-1	0.60	0.40	0.21	0.22	0.95	0.86	0.61	0.56	0.85	0.74
ID-2	0.58	0.43	0.21	0.14	0.94	0.71	0.62	0.61	0.84	0.78
ID-3	0.46	0.19	0.12	0.12	0.77	0.57	0.49	0.38	0.85	0.61
ID-4	0.43	0.23	0.31	0.07	0.91	0.24	0.43	0.41	0.74	0.53
ID-5	0.37	0.20	0.16	0.16	0.73	0.21	0.21	0.39	0.86	0.44
ID-6	0.25	0.02	0.09	0.07	0.43	0.25	0.00	0.14	0.76	0.47

*Kappa values for each subject and the mean kappa for all participating groups*.

### 6.7 Discussion

Two major challenges had to be addressed in this data set. The first one was the influence of eye movement artifacts on the EEG and the second one the generalization of the selected features to be successful on the session-to-session transfer. Like in real conditions the data were from different sessions recorded on different days. Looking at the results it is interesting to compare the performance achieved on data sets of different subjects, while applying the same signal processing algorithms. No method achieved good results on all subjects. Especially the session-to-session transfer could have been a source for the occurred problems. Although we provided training data sets from three different days, 2 training data sets were recorded without feedback and just 1 data set with feedback were given, but of course we wanted to see the performance on online data sets with feedback recorded later on different days. The winning algorithms could foster this problem best, but unfortunately their method needed a 2-s delay to the predicted classification output to achieve a better performance. This approach is very useful if offline classification is performed, but for online control applications such a delay causes a lot of problems for the BCI user.

Like in all the BCI competitions before, the data set and the description will continue to be available on the competition web pagehttp://www.bbci.de/competition/iv/. Other researchers interested in EEG single-trial analysis are welcome to test their algorithms on these data sets and to report their results. To imitate competition conditions, all selections of method, features, and model parameters must be confined to the training sets. However, due to the current availability of the labels of the test data and the publication of thorough analyses of these data, future classification results of the competition data cannot fairly be compared to the original submissions.

## 7 Data Set 3

Data set 3 *Directionally modulated MEG activity* is provided by S. Waldert, C. Braun, H. Preissl, N. Birbaumer, A. Aertsen, and C. Mehring from Freiburg (Germany), Tübingen (Germany), Trento (Italy), and London (UK). It was recorded in a collaboration of the Institute of Biology I, the Bernstein Center Freiburg (both at the University of Freiburg), the MEG-Center and the Institute of Medical Psychology and Behavioral Neurobiology (both University of Tübingen). It can be freely accessed via http://www.bbci.de/competition/iv/ with the only restriction that the present article as well as (Waldert et al., 2008) is referenced upon any publication of results.

### 7.1 Background

Spinal injury patients rank the loss of hand function as one of the most debilitating features of their injury (Anderson, 2004). An intuitive way to realize a brain-machine interface (BMI) is to access the neural cortical activity that controlled natural hand movements and translate this activity into commands that produce equivalent movements of external effectors (e.g., prosthetic arm/hand, computer cursor). Such direct motor BMIs require that kinematic parameters of the movement (e.g., movement direction or velocity) can be inferred from the measured neuronal signals.

**Figure 21:**
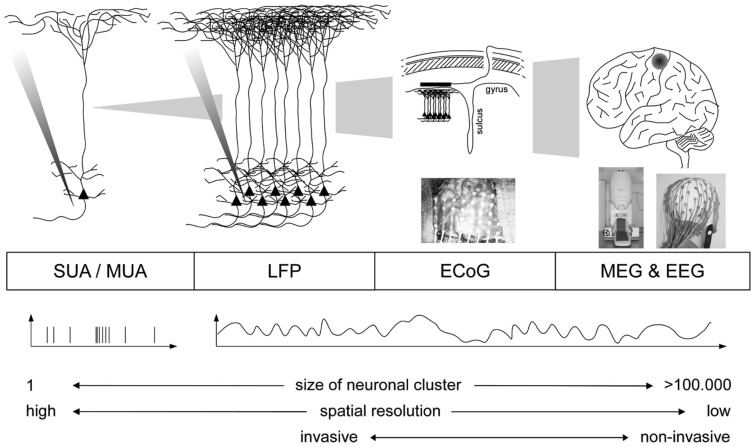
**(Data Set 3)**. Schematic overview of different recording techniques for BMIs (from Waldert et al., 2009 with permission).

Online direct motor BMIs have until recently only been realized using spiking activity [single- (SUA) or multi-unit activity (MUA), e.g., Hochberg et al., 2006; Velliste et al., 2008]. Only in the last decade, it has been shown that not only spiking but also neuronal population activity (Figure 21) is tuned to the direction of hand movements. Tuning of neuronal population signals has been demonstrated in several studies using either (a) invasive recordings (local field potentials, LFP; Mehring et al., 2003) and electrocorticogram (ECoG; Leuthardt et al., 2004; Schalk et al., 2007; Pistohl et al., 2008) or (b) non-invasive recordings (electroencephalogram, EEG; Hammon et al., 2008; Waldert et al., 2008; Bradberry et al., 2010; Lv et al., 2010; Wang and Makeig, 2010) and magnetoencephalogram (MEG; Georgopoulos et al., 2005; Waldert et al., 2008; Bradberry et al., 2009; Wang et al., 2010). Very recently, online direct motor BMI control based on decoding movement direction was realized using MEG (Witte et al., 2010) and ECoG (Milekovic et al., 2012).

Among all these studies, intra-cortical recordings (SUA, MUA, LFP) yield the highest amount of information to be extracted about movement direction (Waldert et al., 2009). However, these signals require the implantation of micro-electrodes into the cortex and long-term stable recording of spiking activity remains a difficult problem. Non-invasive EEG and MEG provide less information, but allow for an easy access to human neural activity without any medical risk for the subject. Obviously, current MEG systems cannot be a basis for real-world direct motor BMI. However, MEG is convenient for BMI training and rehabilitation attempts in patients (e.g., in stroke patients; Buch et al., 2008). In this context, optimized algorithms for inferring kinematic parameters from MEG signals could facilitate BMI training and increase the performance of non-invasive direct motor BMIs. To encourage the development of new algorithms, we contributed to the BCI competition IV a data set containing MEG signals recorded while subjects performed hand/wrist movements in four different directions.

### 7.2 Materials and subject

**Figure 22:**
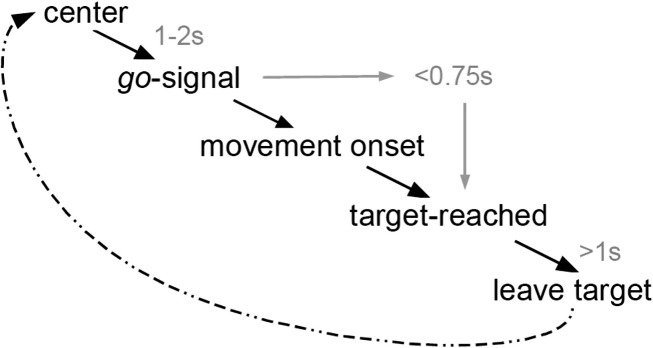
**(Data Set 3)**. Time course of a trial with time constraints (from Waldert et al., 2008 with permission).

The data set contained the signals of 10 MEG sensors (VSM MedTech, Vancouver) above central areas measured at 625 Hz sampling rate during wrist movements of two healthy, right-handed subjects. The subject sat relaxed in an MEG chair, the elbow rested on a pillow to prevent upper arm and shoulder movements, and the head was stabilized by small pillows. The task was to move a joystick from a central resting position toward one of four targets (right, left, forward, backward) using exclusively the right hand and wrist. Movement amplitude was 4.5 cm. In each trial, the target was self-chosen by the subject, i.e., no directional visual cue was provided. Visual trigger signals were presented on a screen in front of the subject to start a trial or to indicate possible time violations. A trial started with the joystick in the center position and the appearance of a gray circle. After a variable delay (1–2 s, Figure 22), the disappearance of the circle indicated the “go” signal (cued movement onset). Then, within 0.75 s the subject had to start the movement and reach the target. For a trial to be valid, the subject also had to rest at the target for at least 1 s. These time constraints allowed for temporal consistency across trials and the hold period at the target prevented interference of in- and outward movements. A red cross was presented continuously for fixation.

### 7.3 Data format and performance criteria

Trials were cut to contain data from 0.4 s before to 0.6 s after movement onset. The signals were band-pass filtered (0.5–100 Hz) and resampled at 400 Hz.

The data were provided as two Matlab “mat”-files, for subject one “S1.mat” and for subject two “S2.mat.” Both files contained the variable *Info*, which provided a detailed description of the data. The second variable, *training_data*, contained 40 labeled trials per movement direction. These 160 trials were provided to train and evaluate the decoding algorithms. The third variable, *test_data*, contained 74 (for S-1) or 73 (for S-2) unlabeled trials in a pseudo-random order. The number of trials per movement direction was unequal but similar. The movement directions of these test trials were not given but had to be predicted from the MEG signals and submitted to the competition. Based on the submitted labels, we calculated the performance of the competitor’s algorithms as the percentage of correctly classified trials (decoding accuracy).

### 7.4 Submissions and algorithms

We received four submissions (ID-1 to ID-4) for this data set. The submission showing best performance was well above chance level for the unlabeled test data. It was submitted by

ID-1: Sepideh Hajipour Sardouie, Mohammad Bagher Shamsollahi. Biomedical Signal and Image Processing Lab (BiSIPL), School of Electrical Engineering, Sharif Univ. of Techn., Tehran, Iran.

The following short summary of the applied algorithms is based on the descriptions provided by the competitors:

ID-1: A comprehensive set of statistical features, frequency-domain features and wavelet coefficients was extracted from 12 channels (10 real channels plus 2 artificial bipolar channels). The number of features was reduced using a supervised algorithm. Then, a genetic algorithm selected features to optimize the classification accuracy. The classifier consisted of a combination of a linear SVM and LDA. Details of this algorithm are published in (Sardouie and Shamsollahi, 2012).

ID-2: First, a low-pass filter (cutoff 8 Hz) was used to filter the time signal. Secondly, the time segment (0–0.5 s) was selected, that is points 160–360. Third, the first three and five principal components of the abs and angle of the 128 FFT of each channel and each sample were used. Then, Fisher discriminant analysis (FDA) was applied to the frequency features to reduce the dimensionality. Fourthly, the signal were subsampled to 20 Hz. Then, FDA was applied to the time features to reduce dimensionality. Finally, Fisher discriminant functions were used for classification using the combination of time and frequency features.

ID-3: Preprocessing unknown. The feature set consisted of statistical, temporal, parametric and wavelet coefficients and was reduced by PCA and a genetic algorithm. The classifier was a linear SVM.

ID-4: First, a low-pass filter (cutoff 8 Hz) was used to filter the time signal. Secondly, the time segment (0–0.5 s) was selected, that is points 160–360. Third, the first three and five principal components of the abs and angle of the 128 FFT of each channel and each sample were used. Then, FDA was applied to reduce dimensionality. Finally, Fisher discriminant functions were used for classification using the frequency feature.

### 7.5 Outcome

**Figure 23:**
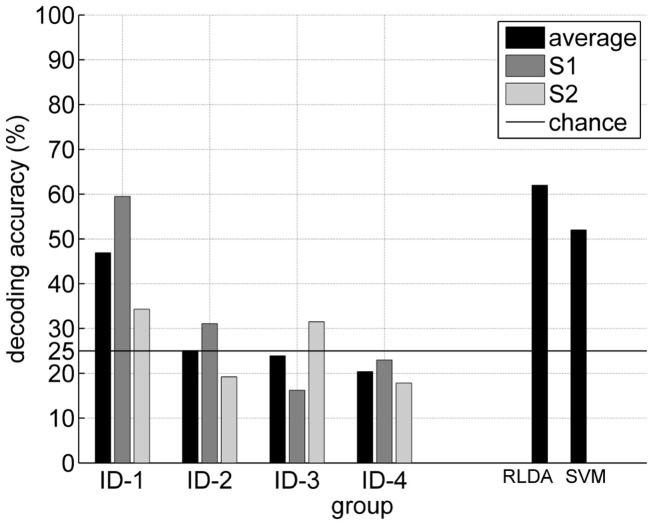
**(Data Set 3)**. Results of the BCI competition IV and, for comparison, the average result of applying a RLDA and linear SVM to the low-pass filtered and resampled activity of the data.

All contributors applied either linear SVM, the linear Fisher discriminant analysis (LDA), or a combination of both. Algorithms mainly differed in feature selection. Three competitors (ID-2/3/4) achieved decoding accuracies around chance level of 25% only for the test data (see Figure 23).

The winner applied a combined linear discriminant analysis (LDA) and linear support vector machine (SVM) on features selected from a large feature set by scattering matrices and a genetic algorithm. The feature set comprised features extracted from the time domain (e.g., AR coefficients, form factor), the frequency domain (e.g., energy in different frequency bands, mean frequency), and the time-frequency domain (wavelet coefficients), but not the low-pass filtered signals that were used in (Waldert et al., 2008). Obtained accuracies on the test data were 59.5% and 34.3% for subjects 1 and 2, respectively, and 46.9% on average.

### 7.6 Discussion

The performance of the competitors algorithms was lower than that of an established decoding algorithm: the application of a regularized linear discriminant analysis (RLDA, also used in Waldert et al., 2008) to the low-pass filtered and resampled signals of the BCI-competition data resulted in a significantly higher average accuracy of 62% (average across both subjects; *p* < 0.01 compared to the competition winner ID-1, Fisher’s exact test). Also a linear SVM using the same low-pass filtered signals yielded a higher average accuracy of 53% (significantly higher than ID-2/3/4 (*p* < 0.01), not significantly higher than ID-1, Fisher’s exact test).

ID-2 and ID-4 obtained much higher accuracies on the training data (98% and 73%) than for the test data, which was classified at chance level. This result indicates that the low accuracies for the test data are due to a poor generalization. Possibly the same reason explains the low accuracy for ID-3. However, the performance on the training data was not available for this group.

Compared to the results of the winning group (ID-1), the higher (RLDA) and equal (SVM) accuracies for the two standard linear classifiers without sophisticated feature selection might be explained by the fact that the low-pass filtered activity – which was used in (Waldert et al., 2008) and which was, due to the applied band-pass filter (0.5–100 Hz, see Data Format), also available in the data set contributed to the BCI competition – was not included in the predefined feature set used by the winning group. It is not clear which decoding accuracies could have been achieved with the algorithm of the competition winner if the low-pass filtered activity were included. Especially this signal component contains substantial information about movement kinematics and provides high performance for decoding of neural population signals: LFP (Mehring et al., 2003; Rickert et al., 2005), ECoG (Schalk et al., 2007; Pistohl et al., 2008; Ball et al., 2009), EEG (Waldert et al., 2008; Bradberry et al., 2010; Lv et al., 2010; Wang and Makeig, 2010), and MEG (Jerbi et al., 2007; Waldert et al., 2008; Bradberry et al., 2009; Wang et al., 2010).

## 8 Data Set 4

Data set 4 *Finger Movements in ECoG* is provided by K. J. Miller and G. Schalk from Seattle and Albany (USA). The data set can be freely assessed via http://www.bbci.de/competition/iv/ with the only restriction that the present article is referenced upon any publication of results.

### 8.1 Motivation

The goal for data set 4 of the BCI competition IV was to infer the flexion of individual fingers from signals recorded from the surface of the brain (electrocorticography, ECoG). Compared to EEG, where a higher spatial blurring prevents the detailed localization in single trial on the finger level, the ECoG signals provide a much higher spatial resolution. This data set contained ECoG signals from three subjects, as well as the time courses of the flexion of each of five fingers. The task in the competition was to use the ECoG signals and flexion information in a training set to predict finger flexion for a provided test set. The performance of that prediction was evaluated by calculating the average correlation coefficient *r* between actual and predicted finger flexion. We received five submissions for this data set. The results of these submissions and recently published studies demonstrate that the timing and degree of finger flexion can be accurately inferred from ECoG in single trials.

Finger flexion is a simple parameter to correlate with an extracted brain state, and thus can serve as a good test bed for algorithm development. There are many potential implications of successful algorithmic decoding of brain states: neural prosthetics, communication devices, handicapped vehicle control (wheelchairs, etc.), and potentially rehabilitation of the brain. The use of motor areas related to hand movements is particularly compelling in this context, because, as an area that is evolutionarily specialized for tool use, it may provide an intuitive basis for controlling prosthetic hands or other manipulandums.

**Figure 24:**
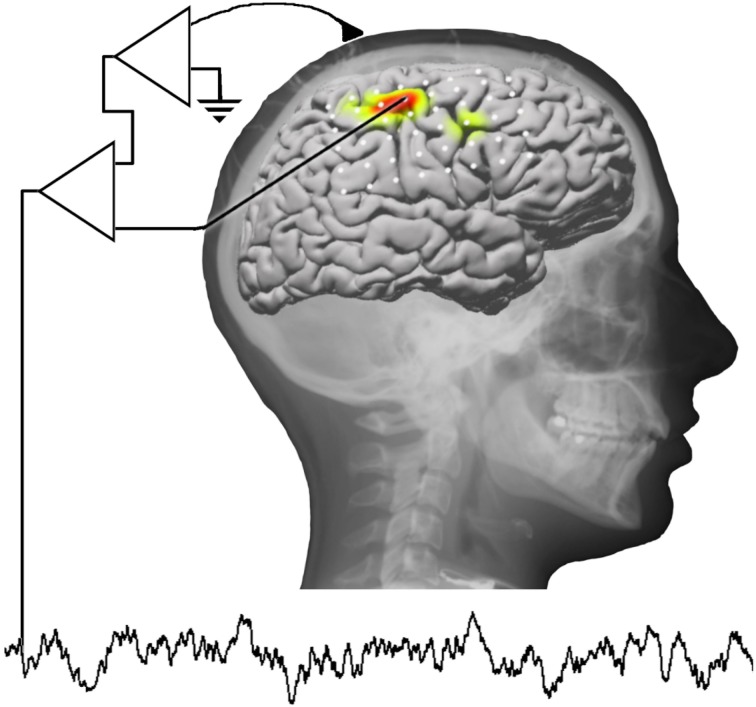
**(Data Set 4)**. The ECoG signals in train_data (time, channel) and test_data (time, channel) were acquired from each electrode with respect to a scalp reference and ground before re-referencing with respect to the common average.

Electrocorticography (ECoG) is the measurement of mesoscale electric potentials (1–5 mm) from the subdural brain surface. In the data set provided for the BCI competition, all three subjects who participated were epileptic patients receiving ECoG monitoring for the localization of seizure foci (Figure 24). In this setting, ECoG has proven to be a powerful tool for brain-computer interfacing (Leuthardt et al., 2004; Schalk et al., 2008), and capable of augmenting activity in the brain (Miller et al., 2010).

**Figure 25:**
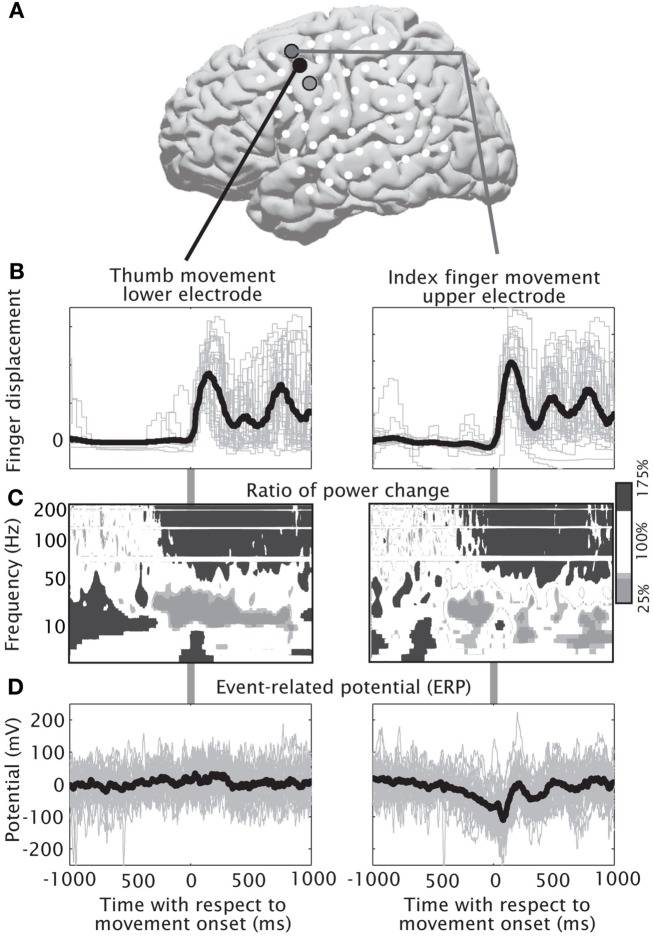
**(Data Set 4 – event-related potential)**. Illustration that the characteristic changes in the power spectral density changes with activity are not due to an reproducible event-related potential shift (ERP). Two adjacent electrodes are shown in **(A)**. One has an ERP, and one does not, but both have the characteristic peri-movement spectral changes. **(B)** Individual (gray) and averaged thumb movement (black, left) or index finger movement (black, right), locked to the first movement from the appropriate movement cue. **(C)** The normalized power spectral density (“PSD”) as a function of time. It demonstrates the classic spectral changes just prior to movement onset for both thumb and index finger. Note that the decrease in power at lower frequencies (α/β/μ range), and the increase in power at higher frequencies (above about 40 Hz) both begin before movement onset. **(D)** Individual and averaged raw potential traces around each of the first movements from appropriate thumb or index finger movement epochs. There is no significant event-related potential (ERP) effect for thumb, but there is for the index finger.

**Figure 26:**
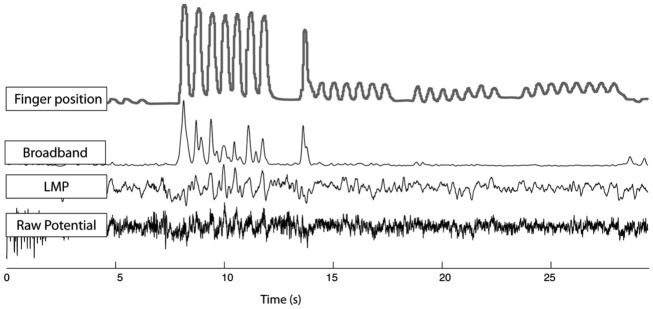
**(Data Set 4)**. Time courses of finger flexion, broadband, LMP, and the raw electric potential. The LMP (Schalk et al., 2007; Kubanek et al., 2009) has been shown to hold information about different motor behaviors. Spectrally broadband change, corresponding to 1/f type change in the electric potential power spectrum (Miller et al., 2009a,b), can be captured as another powerful correlate of motor behavior. By synthesizing different features, more powerful brain-computer interfacing algorithms may be obtained.

**Figure 27:**
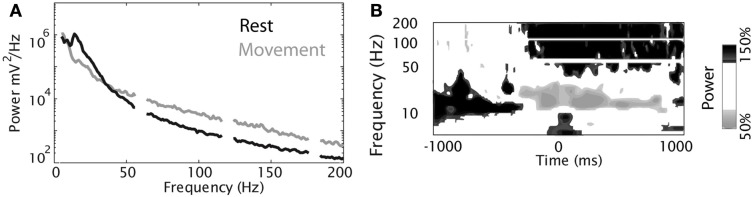
**(Data Set 4)**. Examples of the normalized power spectral density (PSD) of the potential time series around finger flexion. The PSD was calculated from 1 s windows centered at times of maximum flexion and also during rest. **(A)** Mean PSD of index finger movement samples (light trace) and rest samples (black trace). **(B)** Average time-varying PSD (scaled as percentage of mean power at each frequency) with respect to first index finger movement from each movement cue.

**Figure 28:**
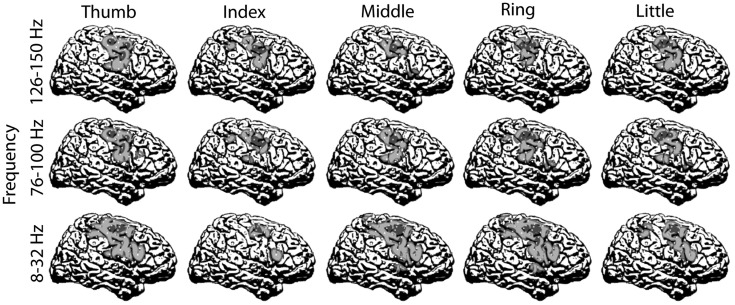
**(Data Set 4)**. Cortical activation maps for movement of different fingers in one subject. The changes in power between 126 and 150 Hz are focused in the classic hand area of the brain. The spatial distribution for 76–100 Hz are nearly identical, as might be expected since both are reflections of the broadband feature highlighted in recent literature (Miller et al., 2009b). Low frequency changes are spatially much more broad, corresponding to fluctuations in the classic motor rhythms. Figure 29 shows that the spatial representations for high frequencies are very different for different finger movement types, within a general hand region. Electrode positions are shown with white dots, and power change with light and dark gray patches on the brain surface.

**Figure 29:**
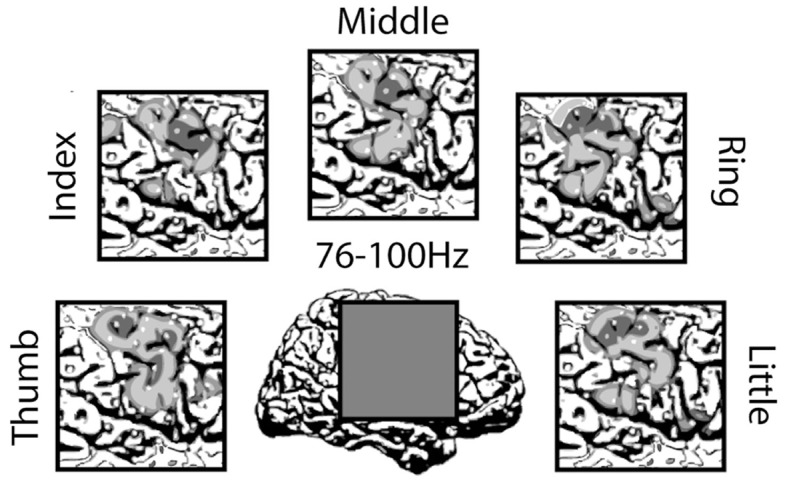
**(Data Set 4)**. A blow-up of the sensorimotor region for high frequencies from Figure 28. Note that this variability across electrodes allows for robust segregation of different finger movements during classification.

**Figure 30:**
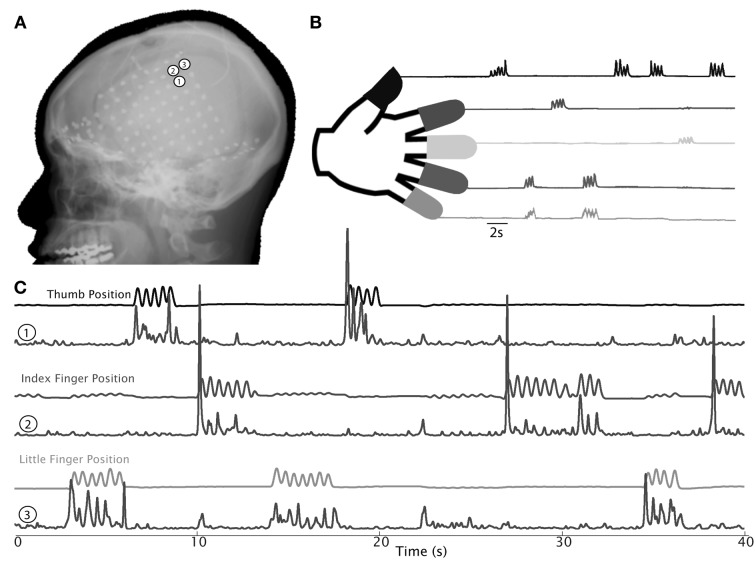
**(Data Set 4)**. Time course of ECoG in adjacent electrodes reveals individual digit representation. **(A)** X-ray of the ECoG array *in situ*, with three electrodes labeled, corresponding to the numbers in **(C)**. **(B)** Flexion time course of each finger. **(C)** Projections of the time-frequency representation to broadband spectral change (Miller et al., 2009b). Each electrode is specifically and strongly correlated with one movement type (r = 0.46 for broadband from electrode 1 with thumb position; r = 0.47 for electrode 2 with index finger; r = 0.29 for electrode 3 with little finger; cross-combinations had a mean correlation of −0.09, indicating light hyperextension of other fingers while flexing the appropriate finger in this subject), over 10 min of continuous data (3.6 × 10^6^ samples).

Several features can be extracted from the ECoG data that may correlate with behavior. Motor-related event-related potentials can be extracted from the raw time series (Figure 25D). A running average of the raw signal, termed the local motor potential (LMP; Schalk et al., 2007) has been shown to be informative about task-related brain activity in motor cortex (Schalk et al., 2007; Kubanek et al., 2009; Figure 26). In addition, frequency-domain features have been shown to robustly capture shifts in behavioral state (Crone et al., 1998a,b; Miller et al., 2007). Shifts in different frequency ranges often have different spatial patterns. There is a characteristic decrease in power at low frequencies and increase in power at high frequencies that accompanies movement (Figure 27). The decreases in low frequency power have spatially broad distributions, and power increases at high frequencies have spatially more confined distributions (Figure 28). Different fingers have spatially different representations on the brain surface, and this can be used to help distinguish which finger might be moving at any particular time (Figures 28–30).

Time-frequency estimates of power change can serve as robust correlates of behavior (Figures 25 and 27). Recent studies have demonstrated that what had been perceived as a spatially focal high frequency phenomenon was really a reflection of a broadband feature, likely corresponding to average firing potential rate of the neuronal population beneath the electrode (Miller et al., 2009a,b). When captured, this broadband feature has been demonstrated to be a robust correlate of finger movement at individual sites in motor cortex (Miller et al., 2009b; Figures 26 and 30).

In this competition, participants used different techniques that capitalized on different aspects of these signals to predict the flexion of individual fingers from the ECoG signals.

### 8.2 Materials and subjects

The three subjects in the data set were epileptic patients at Harborview Hospital in Seattle, Washington. Each patient had electrode grids placed subdurally on the surface of the brain for the purpose of extended clinical monitoring and localization of seizure foci. Each subject gave informed consent to participate in this study, which was approved by the internal review board (IRB) of Harborview Hospital. All patient data have been anonymized according to IRB protocol in accordance with HIPAA regulations.

#### 8.2.1 Experimental paradigm

Signals from the electrode grid were amplified and digitized using Synamps2 amplifiers (Neuroscan, El Paso, TX, USA). The general-purpose BCI system BCI2000 (Schalk et al., 2004) provided visual stimuli to the patient, acquired brain signals from the Synamps2 system, and also recorded the flexion of individual fingers (on the hand contralateral to the implanted grid) using a data glove (Fifth Dimension Technologies, Irvine, CA, USA). BCI2000 stored the brain signals, the timing of stimulus presentation, and the flexion of each of the fingers in a data file. Data files were converted to MATLAB format for this competition. Each patient had subdural electrode arrays (Ad-Tech, Racine, WI, USA) implanted. Each array contained 48–64 platinum electrodes that were configured in 8 × 6 or 8 × 8 arrangements. The electrodes had a diameter of 4 mm (2.3 mm exposed), 1 cm inter-electrode distance, and were embedded in silastic. Electrocorticographic (ECoG) signals (i.e., 62, 48, and 64 channels from subjects 1, 2, and 3, respectively), were acquired with respect to a scalp reference and ground (Figure 24), band-pass filtered between 0.15 and 200 Hz, and sampled at 1000 Hz.

#### 8.2.2 Protocol

The subjects were cued to move a particular finger by displaying the corresponding word (e.g., “thumb”) on a computer monitor placed at the bed-side (Figure 30). Each cue lasted 2 s and was followed by a 2-s rest period during which the screen was blank. During each cue, the subjects typically moved the requested finger 3–5 times. This number varied across subjects and fingers. There were 30 movement stimulus cues for each finger (i.e., a total of 150 cue presentations and about 90–150 flexions of each finger); stimulus cues were interleaved randomly. This experiment lasted 10 min for each subject.

Subsequent offline analyses showed that ring (4th) finger movements were correlated with either middle (3rd) or little (5th) finger movements. Thus, while this ring finger position was included with the training data, it was not used for evaluation.

### 8.3 Data format

The data for each subject was contained in a separate MATLAB file that was named “*subX_comp.mat*” where “*X*” denotes the subject number. Each file contained three variables:

- “*train_data*” – this variable, in time × channels, gave the first 2/3 (6 min, 40 s) of recorded ECoG signals (400,000 samples at 1 kHz sampling rate per channel) from the specified experiment, for every channel.
- “*train_dg*” – this variable, in *time* × *finger* was the first 2/3 (6 min, 40 s) of recorded finger position [thumb – index – middle – ring – little; 400,000 samples (super-sampled to 1 kHz) per finger] for the associated experiment.
- “*test_data*” – this variable, in time × channels, gave the last 1/3 (3 min, 20 s) of recorded ECoG signals (200,000 samples at 1 kHz sampling rate per channel) from the specified experiment, for every channel. These data were used to predict the final 1/3 (3 min, 20 s) of recorded finger position (thumb – index – middle – ring – little) for the associated experiment.

The channel order was scrambled so that the prediction task in the competition was restricted to algorithmic optimizations only.

### 8.4 Challenge

Each participating group submitted three files titled “*sub1_eval*,” “*sub2_eval*,” and “*sub3_eval*, ” corresponding to subjects 1–3, respectively. Each of these contained a single variable, “*eval_dg*,” with dimensions 200,000 × 5:

- “*eval_dg*” – this variable, in time × channels, gave the last 1/3 (3 min, 20s) of predicted finger flexion for each of the five fingers (thumb – index – middle – ring – little) for the associated experiment (200,000 samples per finger).

The evaluation criteria was as follows: for each subject, the received variable “*eval_dg*” was compared with the actual finger positions in “*test_dg*,” which was withheld. We calculated the correlation coefficient *r* between the actual and the predicted finger flexions for each subject and finger. We did not calculate the correlation coefficient for the 4th (ring) finger, because the flexion of this finger was typically correlated with the flexion of the 3rd (middle) or 5th (little) finger. The final score was calculated as the arithmetic mean of the 12 correlation coefficients (4 per subject, 3 subjects). The submission with the highest score won the competition.

### 8.5 Submissions and algorithms

Five groups submitted a contribution (S-1 to S-5), with three of them (S-1, S-2, S-4) showing a performance well above chance level on the unseen test set.

**S-1**: Remi Flamary, Alain Rakotomamonjy, LITIS INSA de Rouen, France
**S-2**: Nanying Liang and Laurent Bougrain, Cortex Team, Research Centre INRIA, Nancy-Grand Est, France
**S-4**: Mathew Salvaris, University of Essex, Colchester, UK

The following short summary of the applied algorithms is based on the descriptions provided by the competitors:

#### S-1

Flamary and Rakotomamojy employed a switching model to predict finger flexion. This method assumed that the output flexion of the fingers is linear and that the transfer function between ECoG signals and finger position depended on an internal state *k* that represents the finger moving (1–5) or no finger moving at all (6). They used ridge regression to compute the transfer function and sparse linear regression to derive the state estimation. In brief, signals were first down-sampled by a factor of 4. The features for the linear transfer functions were obtained with a Savitsky-Golay filter (0.4 s, 3rd order). The features used for the state estimator were AR coefficients computed on a moving window of 300 points. Once the internal state was estimated, finger flexion was computed by multiplying the features at a time *t* by the linear transfer function corresponding to the state *k* at time *t*.

#### S-2

Liang and Bougrain first extracted, from each location, the time-varying activity in three frequency bands: 1–60, 60–100, and 100–200 Hz. Then, the power in each bin was accumulated in 40 ms time bins. The size of the time bin was chosen so that the resulting amplitude modulation feature inputs had the same sampling rate (i.e., 25 Hz) as that of the finger flexion values. Initial evaluations found that each finger flexion was correlated to features from only two or three particular locations. Therefore, features were automatically selected (separately for each finger and subject) using a stepwise feature selection procedure based on the train and validation method (i.e., 2/3 of train data were used for training and 1/3 for validation). The resulting features were then submitted to a Wiener filter with 25 tap-delays (i.e., using the present input and the previous 1-s inputs for predicting the present finger flexion).

#### S-4

Salvaris first re-referenced signals to the common average, and then down-sampled signals to 500 Hz. Bandpower features were extracted by wavelet packets with sym9 wavelet and the average of the time series. Features were then selected using WEKA’s CFS algorithm. The selected features were used to train the SVR algorithm implemented in LibSVM. The parameters for SVR were tuned through 5-fold cross validation. The resulting SVR model was then used to classify the test data.

### 8.6 Results

The goal of this portion of the competition was to predict finger flexion for four of the five fingers on a test set (3 min 20 s) using a classifier that was trained on a training set (6 min 40 s). The fidelity of the prediction was assessed by computing the correlation coefficient between the actual finger flexion values and the submitted finger flexion values. The result of a particular submission was the arithmetic mean of 12 correlation coefficients (i.e., 3 subjects and 4 fingers).

Two of the five submissions achieved particularly strong predictions (see Table 5). Nanying Liang and Laurent Bougrain achieved an average correlation coefficient of 0.46, and thereby won this competition. The runner-up contribution of Flamary and Rakotomamojy performed similarly well with a correlation coefficient of 0.42. Details of the two approaches are described in Flamary and Rakotomamonjy (2012) and Liang and Bougrain (2012).

**Table 5:** **(Data Set 4)**. Performance of the five submissions.

Submission	r
S-2	0.46
S-1	0.42
S-4	0.27
S-3	0.10
S-5	0.05

While it is difficult to assess the difference in performance between the different methods, it is interesting that methods that are similar in simplicity to those used in Schalk et al. (2007) and Kubanek et al. (2009) can reliably and robustly estimate finger flexion from ECoG signals. That being the case, it may also be possible that more sophisticated methods that explicitly incorporate physiological or physical constraints in the computational model might further improve performance.

## 9 Discussion

The BCI competition was created in order to support the development of algorithmic solutions for typical BCI problems. Does it live up to its promise? The following sections attempt to give an answer to the various aspects of this question.

### 9.1 Rest class problem remains a challenge

Moving from an artificial lab situation to the every-day use of a BCI introduces a new challenge: periods of non-control, where a BCI user is voluntarily switching to another (non-BCI) action or is involuntarily distracted from the control interface. The distractor can be another active task (e.g., communication via a different channel, the perception and processing of content, reasoning about a decision to take) or simply taking a rest. The basic problem about rest class detection in general is, that the resting state is not well-defined at all, and thus there is no reliable training data available that can be used to calibrate the BCI system.

In this competition, the detection of such a rest class was challenged with data set 1. The results for this motor imagery data set revealed, that most competitors had problems in correctly identifying time periods of the rest class. Even considering the performance of the competition winner (Figure 6) there remains the wish for further improvement.

### 9.2 Transferability to online BCIs

The winning methods of this or earlier competitions are not necessarily transferable to be used in an online closed-loop BCI system. While the runtimes of algorithms are not a real limitation, non-causal filters and time delays are problematic. As an example, the winning algorithm of data set 2b predicted the class labels quite accurately, but introduced a delay of 2 s during the preprocessing eye artifacts. This is a trade-off that has to be considered for each specific application.

High robustness and generalizability of a winning algorithm is another characteristic, that supports the applicability of an algorithm for the feedback case. Comparable to earlier BCI competitions, again variants of CSP ruled the rankings. Of special interest is the outcome, that the Singapore group has scored exceptionally high for several of the data sets. As the submitted algorithms of this group used similar concepts, this is a strong indicator for robustness.

As some of the winning algorithms of earlier competitions have indeed been adopted into the standard canon for BCI online control, we believe, that this will also happen for some algorithms of the present competition.

### 9.3 Usefulness of synthetic data

The most reliable way of testing new algorithmic ideas for BCI is to implement them in an online experiment, if possible with users matching the target group. But even when testing with healthy users, the testing effort is huge and can not be invested for every change of the algorithmic model.

Synthetic EEG data as presented in data set 1-artificial might offer a partial remedy to this problem. It has proven to be realistic in the sense that the submitted algorithms performed very similar on the synthetic and the real EEG data. As it is cheap to generate a large amount of this data, at least initial algorithmic test beds can be based on it. Precautions, of course, have to be taken in order to avoid that the priors used for the EEG generation are not known or explicitly exploited by the algorithms under test and their creators.

As simulated BCI classifier output has already successfully been applied for the fine-tuning of BCI user interfaces (Quek et al., 2011), the next step is on the horizon: to use simulated EEG that – certainly only to a limited extend – models the user behavior, in order to test BCI systems online in a closed loop.

## 10 Further Topics Concerning Future competitions

Due to the development of the field of BCI, new data analytic problems were identified, that are suitable for addressing them in a BCI competition.

### 10.1 Wireless and dry EEG signals

We currently observe the upcoming of easy-to-mount dry electrode caps as either research prototypes (Popescu et al., 2007; Gargiulo et al., 2010; Luo and Sullivan, 2010; Saab et al., 2011; Zander et al., 2011) or purchasable products (e.g., Sahara dry cap by gTec, Mindset by NeuroSky, or Emotiv cap). As some of them provide wireless transmission protocols, they open up the possibility to monitor the acting brain during real-life situations rather than under artificial lab conditions.

The signals of these dry electrodes, however, currently still suffer from a number of artifacts, which are typically much weaker or not present at all in wet electrode recordings. Examples are inductive artifacts by persons moving in the same room, drifts and saturation effects, or friction artifacts upon electrode movements. While an overall higher noise level of dry electrodes might be difficult to overcome, some artifacts might be alleviated by suitable data processing. A future BCI data competition should thus include a number of dry sensor data sets to determine the most effective approaches.

### 10.2 Non-stationarity

Severe for the use of dry EEG sensors, but not restricted to this signal type, is the problem of non-stationarity in brain signals. In the context of BCI, it is mostly observed during the transition from the initial calibration phase to the online use of a BCI (Shenoy et al., 2006; Sugiyama et al., 2007), but also within periods of online use, where no obvious change of the task or paradigm takes place.

The reasons for non-stationarity in brain data can range from external noise, over effects caused by high dimensionality and robust estimation problems (Sannelli et al., 2008; Abrahamsen and Hansen, 2011; task-unrelated) changes in the background brain activity of BCI users (e.g., due to fatigue or artifacts; Winkler et al., 2011, learning effects or adaptive behavior of the users; Ramsey et al., 2009, or even co-adaptation of users and the BCI system Vidaurre et al., 2011).

Non-stationarity can sometimes be observed even by bare eye in the raw data, where it is present in the form of slow drifts, changes in oscillatory sources, or changes in the noise level of electrodes. If processed with an automatic classification or regression method as in BCI, this processing can be harmed also by subtle bias shifts, covariance drifts or changes of the covariance structure, or even more complex changes of the data distributions.

Although a number of methods have been proposed to mitigate this problem either by finding a global stable subspace for the data representation (Krauledat et al., 2007; Blankertz et al., 2008a; von Bünau et al., 2009; Wojcikiewicz et al., 2011), or by adapting the online processing to compensate for ongoing changes (see Vidaurre and Schlögl, 2008; Blankertz and Vidaurre, 2009; Sannelli et al., 2011) for adaptation in motor-related tasks, and (Dähne et al., 2011) for adaptation in ERP paradigms), it still is the source of major problems in the online use of BCI. This qualifies the problem of non-stationarity for becoming a target in future BCI competitions.

### 10.3 Multimodal signals/hybrid BCIs

Considering the predominant use of non-invasive BCI systems, it is worth to briefly review the development of BCI performance (e.g., in terms of communication rates) over time. On the positive side, new BCI systems based on external stimuli have recently been reported, that employed novel paradigms for auditory (Schreuder et al., 2010, 2011; Höhne et al., 2011) and for visual ERP setups (Liu et al., 2010; Acqualagna and Blankertz, 2011; Schaeff et al., 2011; Tangermann et al., 2011; Treder et al., 2011). They improve over long-used standard stimulation paradigms or can provide solutions for patients that have lost eye gaze control. In contrary, the improvements reported for BCI systems based on motor imagery and ERD/ERS effects have been slower over the last years, despite of a drastic initial performance boost which was made possible by the introduction of machine learning methods (Blankertz et al., 2003, 2011; Schröder et al., 2003).

The next boost of BCI performance can be expected for paradigms, that are able to combine independent information from different sources in order to improve the BCI control quality over the level of a traditional single-source BCI. In an ERP setup, such approaches could combine stimuli of different sensory modalities (Aloise et al., 2007). In motor imagery, the use of ERD/ERS effects together with slower motor-related potentials (Dornhege et al., 2004) can increase information rates. Abstracting this concept to the next level, EEG signals could be combined with other brain signal sources like fNIRS (Fazli et al., 2012), with non-neural but physiological signals (e.g., heart rate variability, galvanic skin resistance, pupil dilation, etc.) or in a hybrid setup (Millán et al., 2010; Pfurtscheller et al., 2010; Müller-Putz et al., 2011) e.g., in combination with non-BCI assistive technology.

We currently observe an expansion of BCI technology to other fields. As it gives access to the real-time monitoring of mental states (Müller et al., 2008), it is interesting for neuro-ergonomic interface- and product design (Blankertz et al., 2010; Porbadnigk et al., 2010). Furthermore it starts becoming a tool for the neurosciences, where the use of multiple sources of information is an inviting possibility. All these fields can profit from processing methods, that are capable of linking brain data with behavioral data or with non-neural physiological signal types.

The challenge in processing signals from multiple sources is to represent, combine and converge information in a way, that is independent of different sampling rates (Bießmann et al., 2009; Biessmann et al. 2011), SNR-levels or varying levels of non-stationarity. It is a great challenge with multiple facets. A next BCI competition could contribute to the exploration of at least a few of these aspects.

### Performance baseline for participation

The results of competition IV and the three past competitions have shown, that the number of entries per data set varies to a large extent, probably due to differing levels of effort that have to be invested. Participants tend to submit more entries for standard learning problems, e.g., classification problems where the percentage of correct classifications is the metric of choice. Non-standard learning problems, even though representing important problems in the field of BCI, tend to gain less attention.

As the success of a participant is finally expressed as a rank among all submitted entries, the small sample ranking can potentially be misleading with respect to the overall quality of even the best-ranked entry. For this reason, it is planned to introduce a performance threshold in future BCI competitions. It will be determined based on the test data. All entries have to pass this threshold before they can enter the official ranking. The threshold is to be defined by the data issuing group and should represent the state-of-the-art performance that can be gained with established analysis methods. The threshold is published together with the performance metric and with a short description of the standard method that leads to this performance.

We think that this action will contribute toward assessing the absolute quality of a competition entry rather than the relative quality only. On the long run the introduction of a threshold can increase the perceived reliability of novel methods brought to the BCI community via a BCI competition, and speed up their adoption by BCI practitioners.

## Conflict of Interest Statement

The authors declare that the research was conducted in the absence of any commercial or financial relationships that could be construed as a potential conflict of interest.

## Footnotes

^1^The decomposition is not unique but any will do.

^2^One test data set is distributed from the beginning of the competition to enable participants to test their program and to ensure that it produces the desired output.

^3^One test data set is distributed from the beginning of the competition to enable participants to test their program and to ensure that it produces the desired output.

## A Appendix

### A.1 Data set 2a

All files are listed in Table A1. Note that the test sets will be made available after the deadline of the competition (except for one file from subject A01, which serves as an example). The GDF files can be loaded using the open-source toolbox BioSig, available for free at http://biosig.sourceforge.net/. There are versions for Octave^4^/FreeMat^5^/MATLAB^6^ as well as a library for C/C++.

**Table A1:** **(Data Set 2a)**. List of all files contained in the data set 2a, the striked out test data sets were provided only after the deadline of the competition.

ID	Training	Test
1	A01T.gdf	A01E.gdf
2	A02T.gdf	A02E.gdf
3	A03T.gdf	A03E.gdf
4	A04T.gdf	A04E.gdf
5	A05T.gdf	A05E.gdf
6	A06T.gdf	A06E.gdf
7	A07T.gdf	A07E.gdf
8	A08T.gdf	A08E.gdf
9	A09T.gdf	A09E.gdf

*Note that due to technical problems the EOG block is shorter for subject A04T and contains only the eye movement condition*.

**Table A2:** **(Data Set 2a)**. List of event types in data set 2a (the first column contains decimal values and the second hexadecimal values).

Event type		Description
276	0 × 0114	Idling EEG (eyes open)
277	0 × 0115	Idling EEG (eyes closed)
768	0 × 0300	Start of a trial
769	0 × 0301	Cue onset left (class 1)
770	0 × 0302	Cue onset right (class 2)
771	0 × 0303	Cue onset foot (class 3)
772	0 × 0304	Cue onset tongue (class 4)
783	0 × 030F	Cue unknown
1023	0 × 03FF	Rejected trial
1072	0 × 0430	Eye movements
32766	0 × 7FFE	Start of a new run

**Table A3:** **(Data Set 2b)**. List of all files contained in the data set 2b, the striked out test data sets will be provided after the deadline of the competition.

ID	Training	Test
1	B0101T, B0102T, B0103T	B0104E, B0105E
2	B0201T, B0202T, B0203T	B0204E, B0205E
3	B0301T, B0302T, B0303T	B0304E, B0305E
4	B0401T, B0402T, B0403T	B0404E, B0405E
5	B0501T, B0502T, B0503T	B0504E, B0505E
6	B0601T, B0602T, B0603T	B0604E, B0605E
7	B0701T, B0702T, B0703T	B0704E, B0705E
8	B0801T, B0802T, B0803T	B0804E, B0805E
9	B0901T, B0902T, B0903T	B0904E, B0905E

*The first two sessions (…01T, …02T) contain training data without feedback, and the last three sessions (…03T, …04E, …05E) with smiley feedback. Note: Due to technical problems no recording for EOG estimation (eyes open, closed, eye movements) exists in session B0102T and B0504E*.

A GDF file can be loaded with the BioSig toolbox with the following command in Octave/FreeMat/MATLAB (for C/C++, the corresponding function HDRTYPE * sopen and size_t sread must be called):

[s,h] = sload(’AO1T.gdf’);

Note that the runs are separated by 100 missing values, which are encoded as not-a-numbers (NaN) by default. Alternatively, this behavior can be turned off and the missing values will be encoded as the negative maximum values as stored in the file with:

[s,h] = sload(’AO1T.gdf’,0,

’OVERFLOWDETECTION:OFF’);

The workspace will then contain two variables, namely the signals s and a header structure h. The signal variable contains 25 channels (the first 22 are EEG and the last 3 are EOG signals). The header structure contains event information that describes the structure of the data over time. The following fields provide important information for the evaluation of this data set:

h.EVENT.TYP
h.EVENT.POS
h.EVENT.DUR

The position of an event in samples is contained in h.EVENT.POS. The corresponding type can be found in h.EVENT.TYP, and the duration of that particular event is stored in h.EVENT.DUR. The types used in this data set are described in Table A2 (hexadecimal values, decimal notation in parentheses). Note that the class labels (i.e., 1, 2, 3, 4 corresponding to event types 769, 770, 771, 772) are only provided for the training data and not for the testing data.

The trials containing artifacts as scored by experts are marked as events with the type 1023. In addition, h.ArtifactSelection contains a list of all trials, with 0 corresponding to a clean trial and 1 corresponding to a trial containing an artifact.

SigViewer 0.2 (or higher) can be used to view and annotate GDF files. SigViewer is available at http://sigviewer.sourceforge.net/.

### A.2 Data set 2b

All files are listed in Table A3. The GDF files can be loaded using the open-source toolbox BioSig, available for free at http://biosig.sourceforge.net/. There are versions for Octave^7^/MATLAB^8^ as well as a library for C/C++.

A GDF file can be loaded with the BioSig toolbox with the following command in Octave/MATLAB (for C/C++, the corresponding function HDRTYPE* sopen and size_t sread must be called):

[s,h] = sload(’AO1T.gdf’);

Note that the runs are separated by 100 missing values, which are encoded as not-a-numbers (NaN) by default. Alternatively, this behavior can be turned off and the missing values will be encoded as the negative maximum values as stored in the file with:

[s,h] = sload(’AO1T.gdf’,0,

’OVERFLOWDETECTION:OFF’);

The workspace will then contain two variables, namely the signals s and the header structure h. The signal variable contains 6 channels (the first 3 are EEG and the last 3 are EOG signals). The header structure contains event information that describes the structure of the data over time. The following fields provide important information for the evaluation of this data set:

h.EVENT.TYP
h.EVENT.POS
h.EVENT.DUR

The position of an event in samples is contained in h.EVENT.POS. The corresponding type can be found in h.EVENT.TYP, and the duration of that particular event is stored in h.EVENT.DUR. The types used in this data set are described in Table A4 (hexadecimal values, decimal notation in parentheses). Note that the class labels (i.e., 1 and 2, corresponding to event types 769 and 770) are only provided for the training data and not for the testing data.

**Table A4:** **(Data Set 2b)**. List of event types in data set 2b (the first column contains decimal values and the second hexadecimal values).

Event type		Description
276	0 × 0114	Idling EEG (eyes open)
277	0 × 0115	Idling EEG (eyes closed)
768	0 × 0300	Start of a trial
769	0 × 0301	Cue onset left (class 1)
770	0 × 0302	Cue onset right (class 2)
781	0 × 030D	BCI feedback (continuous)
783	0 × 030F	Cue unknown
1023	0 × 03FF	Rejected trial
1077	0 × 0435	Horizontal eye movement
1078	0 × 0436	Vertical eye movement
1079	0 × 0437	Eye rotation
1081	0 × 0439	Eye blinks
32766	0 × 7FFE	Start of a new run

The trials containing artifacts as scored by experts are marked as events with the type 1023. In addition, h.ArtifactSelection contains a list of all trials, with 0 corresponding to a clean trial and 1corresponding to a trial containing an artifact.

In order to view the GDF files, the viewing and scoring application SigViewer v0.2 or higher (part of BioSig) can be used.
